# BCDCNN: breast cancer deep convolutional neural network for breast cancer detection using MRI images

**DOI:** 10.1038/s41598-025-09974-0

**Published:** 2025-08-08

**Authors:** D. E. Martina Jaincy, V. Pattabiraman

**Affiliations:** School of Computer Science and Engineering, Vellore Institute of Technology, Chennai, Tamil Nadu India

**Keywords:** Breast cancer detection (BCD), Adaptive kalman filter (AKF), Pyramid scene parsing network (PSPNet), Jellyfsh search optimizer (JSO), Deep convolutional neural networks

## Abstract

Breast cancer (BC) is a kind of cancer that is created from the cells in breast tissue. This is a primary cancer that occurs in women. Earlier identification of BC is significant in the treatment process. To lessen unwanted biopsies, Magnetic Resonance Imaging (MRI) is utilized for diagnosing BC nowadays. MRI is the most recommended examination to detect and monitor BC and explain lesion areas as it has a better ability for soft tissue imaging. Even though, it is a time-consuming procedure and requires skilled radiologists. Here, Breast Cancer Deep Convolutional Neural Network (BCDCNN) is presented for Breast Cancer Detection (BCD) using MRI images. At first, the input image is taken from the database and subjected to a pre-processing segment. Adaptive Kalman filter (AKF) is utilized to execute the pre-processing phase. Thereafter, cancer area segmentation is conducted on filtered images by Pyramid Scene Parsing Network (PSPNet). To improve segmentation accuracy and adapt to complex tumor boundaries, PSPNet is optimized using the Jellyfish Search Optimizer (JSO). It is a recent nature-inspired metaheuristic that converges to an optimal solution in fewer iterations compared to conventional methods. Then, image augmentation is performed that includes augmentation techniques namely rotation, random erasing and slipping. Afterwards, feature extraction is done and finally, BCD is conducted employing BCDCNN, wherein the loss function is newly designed based on an adaptive error similarity. It improves the overall performance by dynamically emphasizing samples with ambiguous predictions, enabling the model to focus more on diagnostically challenging cases and enhancing its discriminative capability. Furthermore, BCDCNN acquired 90.2% of accuracy, 90.6% of sensitivity and 90.9% of specificity. The proposed method not only demonstrates strong classification performance but also holds promising potential for real-world clinical application in early and accurate breast cancer diagnosis.

**Supplementary Information:**

The online version contains supplementary material available at 10.1038/s41598-025-09974-0.

## Introduction

BC is the most common cancer among women, consisting of about 23% of every female cancer worldwide^1^. In Western countries, many women are affected by BC in their lifetime. BC is a second vital cause of death among women in most countries. Earlier detection is crucial to enhanced subsistence and the total prognosis is linked directly to a phase of disease during diagnosis. Mammography is one of the helpful tools that can detect BC in its earlier phase before bodily symptoms occur. As the footnote, screening mammography is the only examination that is revealed to decrease fatalities owing to BC. The screening process with mammography is connected with a 16% to 40% relative decrease in BC mortality rate amongst women aged between 40 to 74 years^2^. However, cancer can be lost in mammography, especially in women having denser breasts^3^. The cancer tissues with high intensities of pixels are simply detected more than other breast areas^4^. For decreasing false negative diagnoses in mammography, a biopsy is suggested for lesions with more than 2% possibility of having suspicious malignant cancers^5^ and amongst them, less than 30% are identified as malignancy^6^.

The breast MRI is utilized for screening cancer in women with high risk, to phase known cancer and for evaluating treatment responsiveness systemic therapy^7,8^. To decrease unwanted biopsies, currently, MRI is utilized for diagnosing BC^9^ as it has a better ability for soft tissue imaging and is the most sensible method in predicting breast-related diseases, yet, it does not comprise probably hazardous radiation^10^. Diagnostic understanding of the breast MRI is a time-consuming chore, frequently involved not only in the assessment of existing imaging investigation but also comparable with numerous previous imaging tests. Since the temporal and spatial resolution of the dynamic contrast-enhanced (DCE) breast MRI remains to rise, radiologists of breast imaging have worked with reviews about numerous images of individual patients^11^. In current years, computer-assisted methods have been enhanced for detecting irregular lesions as well as identifying tissue attributes in clinical images^6,12^. BC clinical imaging can be utilized for viewing within the human body as it is referred to non-invasive technique to assist doctors for diagnosis and treatment^4^.

The problems experienced by MRI are resolved using machine learning (ML) as well as deep learning (DL). DL and ML methods are successful while testing and training data are from similar feature spaces as well as having similar frequency^13^. When a pattern alters, most of the quantitative data in approaches must be reconstructed from down up utilizing newly obtained classification techniques^13,14^. Attaining the necessary training data and designing systems in clinical applications like breast imaging is complicated^14^. As an outcome, it is better to decrease the necessity for a task required to obtain training data. In such criteria, it should be preferred for fine-tuning from one take to another^15^. DL is a promising method to resolve issues that remain in artificial intelligence (AI) society^16,17^. In contrast to conventional ML techniques that include linear regression, Naïve Bayes classifier, support vector machine (SVM) and logistic regression, DL^18^ approaches employ numerous deep layers of the perceptions, which capture low as well as high-level depictions of data^19,20^. DCNN has lately originated as strong DL approaches that satisfy or else exceed the performance level of radiologists in various well-defined as well as repeated image elucidation chores^11,21^.

The foremost goal is to design BCDCNN for BCD using MRI images. The proposed BCDCNN framework effectively addresses several challenges associated with traditional breast cancer detection methods:

Manual MRI interpretation is time-consuming and requires expert radiologists. BCDCNN automates this process by using a deep convolutional neural network that can quickly and accurately detect breast cancer regions, reducing the workload on clinicians and minimizing human error. Also, MRI images often contain noise and complex tissue structures. The AKF used in the pre-processing phase enhances image quality by reducing noise. Segmenting cancerous regions accurately is critical but challenging due to irregular tumor shapes and varying sizes. The use of PSPNet, optimized by the JSO, enables accurate segmentation by effectively tuning the network parameters to capture multi-scale contextual information. Medical imaging datasets are limited and imbalanced, causing overfitting in models. By applying image augmentation techniques, the method artificially expands the training data diversity, avoiding overfitting issues. The extraction of multiple feature types ensures comprehensive characterization of breast tissue, which is vital for distinguishing benign from malignant regions. The novel adaptive error similarity-based loss function dynamically emphasizes ambiguous or hard-to-classify samples during training. This helps the network focus learning on challenging cases, improving performance and reducing false negatives.

The main contribution of the proposed method is:

- **Proposed BCDCNN for BCD:** Earlier detection of BC decreases mortality risks by offering the best option of finding appropriate treatment and a new method is developed for BCD.
- The segmentation is done by PSPNet, which is optimized by the JSO.
- BCD is conducted employing BCDCNN, wherein the loss function is newly designed based on an adaptive error similarity

The segments beneath are arranged as: Traditional approaches reviewed are elucidated in section "Literature survey", BCDCNN methodology is described in section "Proposed BCDCNN for BCD", BCDCNN outcomes are described in section "Results and discussion" and section conclusion ends with the conclusion of BCDCNN.

## Literature survey

Nasir et al.^15^ presented AlexNet for the prediction of BC that could be utilized in the clinical field for preventing unwanted treatment and biopsies, but it did not assess the method by fusing diverse datasets. Yurttakal et al.^6^ introduced a Convolutional Neural Network (CNN) for characterizing benign or malignant tumors utilizing MRI images. It was capable of distinguishing benign and malignant cancers, even while cancer biologic feature reflects variations. However, the time consumption of the model was high. Eskreis-Winkler et al.^11^ designed DL for identifying cancer-comprising axial slices on breast MRI images. This method saved the radiologist’s time, though it did not expand the database to include every breast MRI. Daimiel Naranjo et al.^22^ developed Gaussian SVM for diagnosing BC. It has the potential to serve as a decision-assisting tool to decrease unwanted biopsies in the benign BC. Nevertheless, this technique failed as it only included the breast lesions with a median pixel dimension lesser than for tumors.

Zheng et al.^4^ devised DL assisted Efficient Adaboost Algorithm (DLA-EABA) for BCD. It obtained higher accuracy in the detection of BC mass and increased the survival rate of patients, but it failed to consider computational time. Onishi et al.^23^ presented ultrafast dynamic contrast-enhanced magnetic resonance imaging (DCE-MRI) for predicting BC. This method revealed a stronger association with few BC attributes particularly histopathology as well as molecular subtypes. However, it did not generate the prognostic imaging markers of BC. Hu et al.^24^ developed multiparametric magnetic resonance imaging (mpMRI) for diagnosing BC. This approach was computationally effective and it does not want intensive image pre-processing, but still failed to execute a validation on the independent databases for investigating the robustness of the methodology. Choi et al.^19^ designed a CNN-based model to predict BC. It had the potential to enhance the diagnosis accuracy of various real-time medical applications. However, the dataset utilized was very small and a substantial quantity of data was required to obtain the best outcomes. Rajasekhar Yadav and Vaegae Naveen Kumar^20^ implemented a Fractional Order Convolutional Neural Network (Frac-CNN), which was optimized by Particle Swarm Optimization (PSO) for BCD. This model captured intricate patterns in mammograms, which enhanced the detection accuracy. However, high false-negative and false-positive rates were the major drawbacks of the model. Anas Bilal et al.^25^ established a quantum-inspired binary Grey Wolf Optimizer SqueezeNet and Support Vector Machines (Q-BGWO-SQSVM) for BCD. Here, the best SVM parameters were selected by Q-BGWO. This model was utilized for reliable and accurate early detection of breast cancer. However, it required high processing requirements.

The summary of the literature survey is presented in Table 1.

**Table 1:** Summary of the literature survey.

Reference	Method	Advantages	Disadvantages
Nasir et al.^15^	AlexNet	Prevent unwanted treatment and biopsies	It did not assess the method by fusing diverse datasets
Yurttakal et al.^6^	CNN	Capable to distinguish benign and malignant cancers, even while cancer biologic feature reflects variations	Time consumption of the model was high
Eskreis-Winkler et al.^11^	DL	Saved radiologist’s time	It did not expand the database for including every breast MRI
Daimiel Naranjo et al.^22^	Gaussian SVM	Decrease unwanted biopsies in the benign BC	It only included the breast lesions with median pixel dimension lesser than for tumors
Zheng et al.^4^	DLA-EABA	Obtained higher accuracy in detection of BC mass and increased survival rate	Failed to consider computational time
Onishi et al.^23^	Ultrafast DCE-MRI	Stronger association with few BC attributes as well as molecular subtypes	It did not generate the prognostic imaging markers of BC
Hu et al.^24^	mpMRI	Computationally effective and it does not want intensive image pre-processing	Failed to execute a validation on the independent databases
Choi et al.^19^	CNN-based model	Potential for enhancing the diagnosis accuracy of various real-time medical applications	The dataset utilized was very small and substantial quantity of data was required
Rajasekhar Yadav and Vaegae Naveen Kumar^20^	PSO-Frac-CNN	Captured intricate patterns, which enhanced the detection accuracy	High false-negative and false-positive rates were the major drawbacks
Anas Bilal et al.^25^	Q-BGWO-SQSVM	Reliable and accurate early detection of breast cancer	It required high processing requirements

### Challenges

The conventional methods reviewed for BCD experienced some drawbacks that are elucidated as follows.

- The model designed in^15^ for BCD was proven as rapid and highly effective in predicting BC, but still, it failed to apply fuzzed ML concepts to data for getting more precise as well as promising outcomes.
- In^6^, the CNN system automatically characterized and classified several dimensions of masses with the assistance of computer assessment without the necessity for the biopsy even though it only utilized pixel information for designing a structure.
- To detect BC, the DL method developed in^11^ was supportive during non-systematic image viewing. However, it did not test and train on the multicenter breast MRI to enhance the generalizability of an approach.
- Gaussian SVM presented in^22^ facilitated precise BC diagnosis when decreasing needless benign breast biopsies, though this approach was not validated in large multicenter studies.
- Across the globe, BC is the most prevailing cancer and it is the second crucial cause of death of women. Thus, diagnosing and prevention tools are necessary to treat BC at the starting stages. However, BC classification is a clinical technique that offers scientists and researchers serious challenges.

## Proposed BCDCNN for BCD

**Fig. 1:**
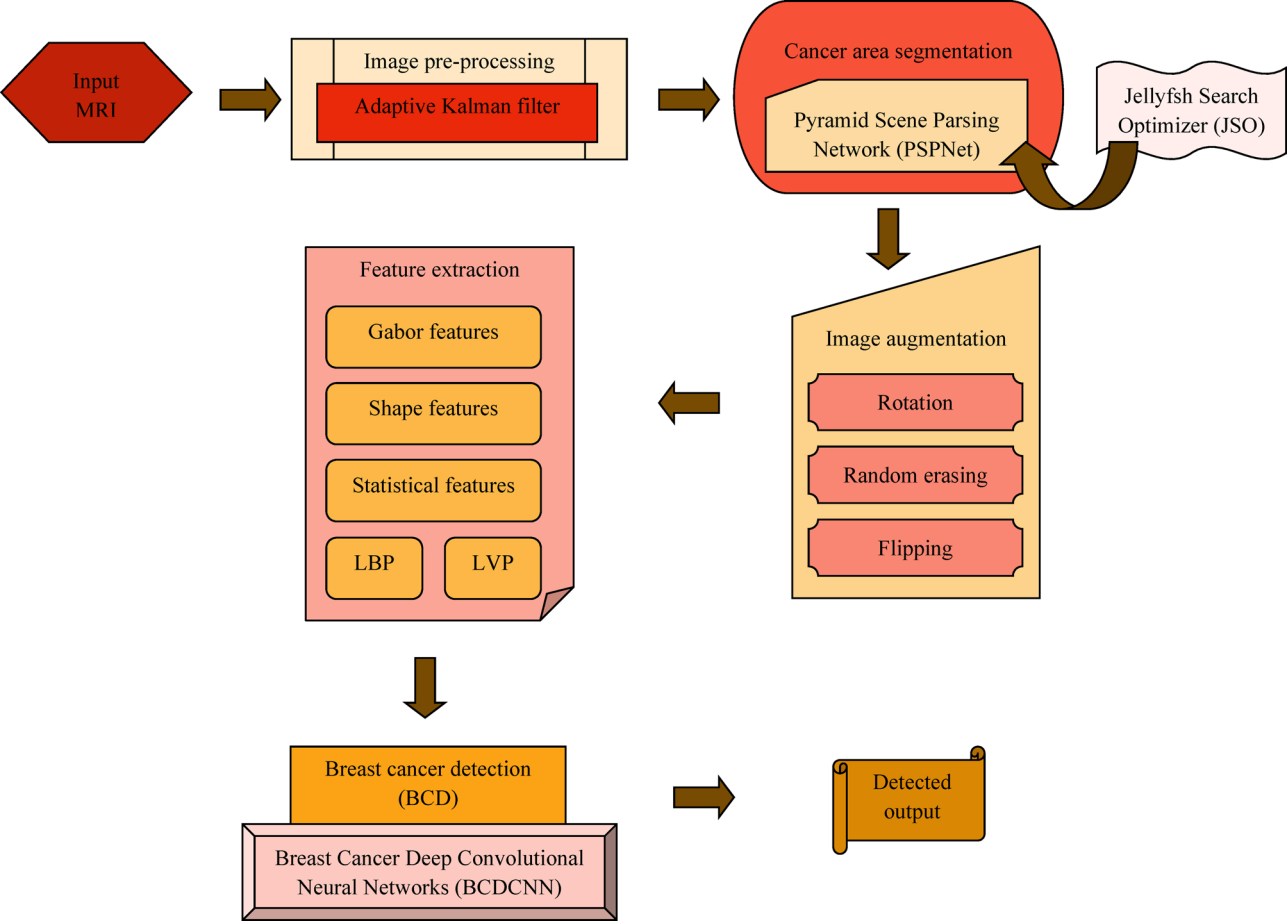
Architecture of the proposed BCDCNN for BCD.

BC refers to an older kind of cancer that affects humans. This cancer disease has been investigated and studied to avoid results caused by it, even though this disease is considered as most deadly disease every time. Here, BCDNN is presented newly to perform BCD. At first, the MRI image considered is given to the pre-processing stage. The pre-processing of the input image is executed by AKF. Afterwards, cancer area segmentation is done employing PSPNet and it is tuned by JSO. Thereafter, image augmentation is done by concerning augmentation methods like flipping, random erasing and rotation. Then, features like LBP, statistical features, Gabor features, LVP and shape features are extracted. At last, BCD is done using BCDCNN, wherein the loss function is newly designed based on adaptive error similarity. The visual representation of BCDCNN for BCD is exposed in Fig. 1.

### Input MRI image acquisition

For performing BCD, input MRI image is taken from datasets specified in^26,27^, which can be illustrated as,

1 $$C = \left\{ {C_{1} ,C_{2} ,...C_{b} ...C_{o} } \right\}$$

where *b*th input MRI image is signified as $C_{b}$ and $C_{o}$ indicates total MRI images in the dataset.

### Pre-processing utilizing AKF

Pre-processing of an image has impressive positive consequences on the outcomes of image assessments. An image pre-processing is similar to the mathematical formulation of dataset normalization, which is the normal phase in various feature descriptor techniques. Moreover, this phase eliminates noises from input images. Here, pre-processing of MRI images is conducted utilizing AKF by considering $C_{b}$ as input.

AKF^28^ is introduced to overcome the disadvantages of the actual Kalman filter method. The AKF is used due to its effectiveness in reducing noise while preserving important image features, which is critical for enhancing MRI image clarity before segmentation. For AKF, a measurement variance $\varsigma_{o}$ has a vital role. A measurement variance value is adjusted for compensating the occurrence of system error or outlying image pixels. The concept is to assign image pixels that are not consistent with a system to random noise by increasing measurement variance artificially, thus image pixels are not utilized for corrupting parameter estimates. A variance of measurement noise is computed by,

2 $$\varsigma_{o} = \left( {1/\lambda } \right)\,\left( {\sum\limits_{\partial = 1}^{\lambda } {\rlap{--} \nu_{o - \partial } \bullet \rlap{--} \nu_{o - \partial } } } \right) - \rlap{--} h_{o}^{{\rlap{--} T}} \bullet \rlap{--} P_{o} \bullet \rlap{--} h_{o}$$

Here, $\lambda$ indicates the count of pixels related to the pre-determined smoothing window, $\partial$ implies index, $\rlap{--} P_{o}$ signifies covariance matrix, $\rlap{--} h_{o}^{{\rlap{--} T}}$ symbolizes measurement function vector whereas $o$ denotes the count of values processed. Also, $K_{b}$ specifies filtered images utilizing an adaptive Kalman filter.

### Cancer area segmentation using PSPNet

The most crucial and vast phase in image processing is image segmentation. This phase is a significant component towards successful assessments of images and pattern recognition. Image segmentation is the group of processes utilized for dividing screened images into various areas. In clinical images, image segmentation has an essential part in segmenting suitable tissues from the background. Here, cancer area segmentation refers to the segmentation of areas covered by malignant tissues. In this work, the cancer area is segmented employing PSPNet which is tuned by JSO. The cancer area segmentation is performed by using filtered image $K_{b}$ as input.

#### Architecture of PSPNet

PSPNet^29,30^ is a multi-scale network that applies a pyramid pooling module to the semantic segmentation field. Thus, it can learn better about global contextual information of scenes as well as efficiently enhance segmentation accuracy. This network has obtained better outcomes in existing rank lists of semantic segmentation. The PSPNet presents rich context details to semantic segmentation and acquires background previously.

In the multi-layer CNN, a receptive field dimension indirectly identifies the degree of utilizing image contextual information. Even though ResNet efficiently expands a receptive field by means of whole convolution as well as additional feature maps, with the deepening of level and increasing the depth of the network, the real receptive field is much less than the theoretical receptive field. A spatial pyramid pooling system efficiently reduces this issue by utilizing pooling at diverse scales and also, and it can broaden the real receptive field in the network. The PSPNet efficiently make utilization of this benefit.

**Fig. 2:**
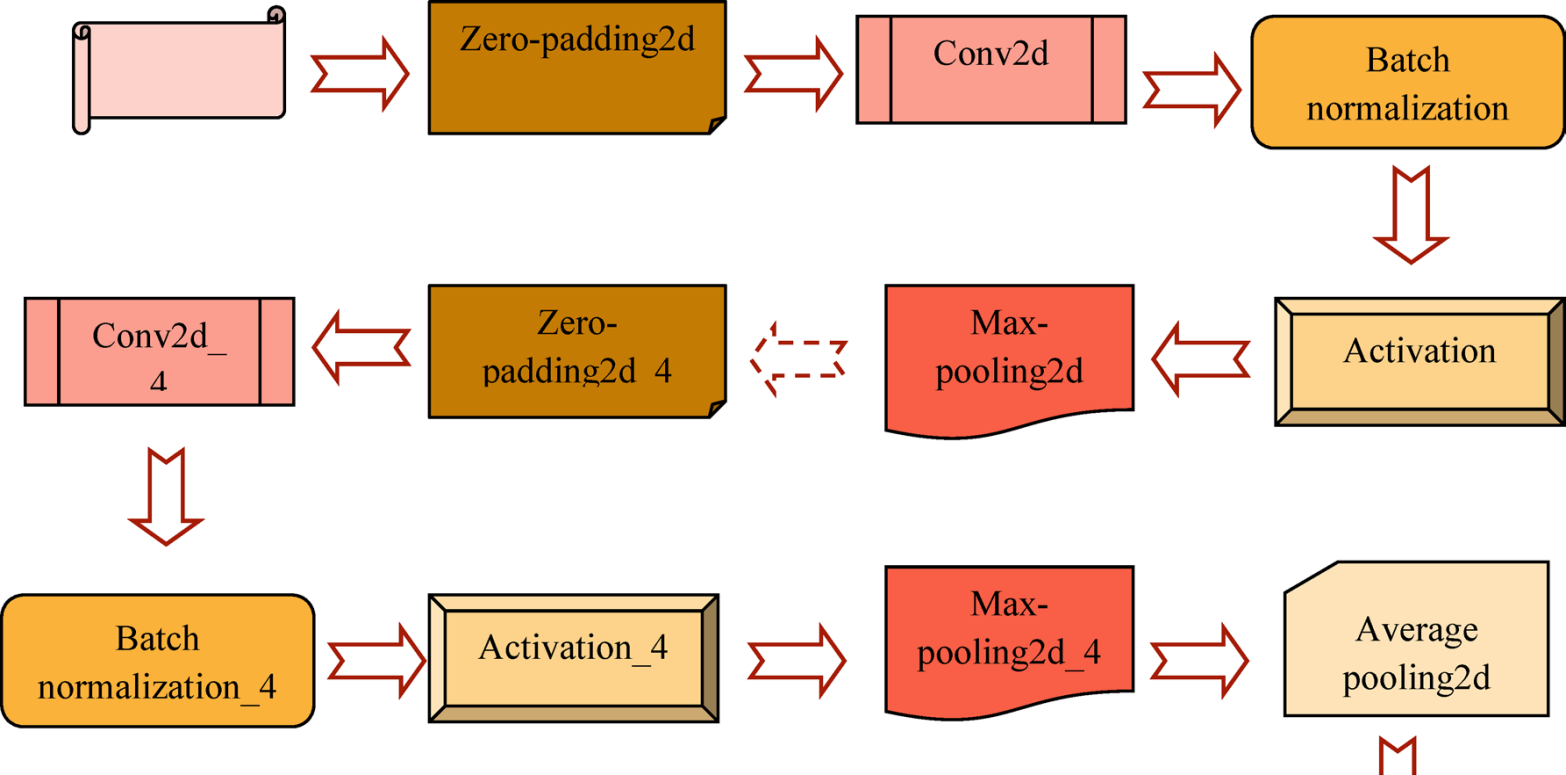
Structure of PSPNet.

Initially, the feature map is acquired by input image features extraction through the steps of CNN. Generally, a CNN network on the basis of ResNet architecture is employed. Also, the pyramid adaptive average pooling module is utilized for capturing the features of diverse subareas at various partition scales and subarea features are upsampled to a similar size as global features preceding pooling and the CONCAT function is executed with global features preceding pooling, thus existing feature map comprises local as well as global features, improving the information of feature map. Finally, the last prediction outcomes are attained by certain upsampling and convolution operations. In the pyramid pooling module, the scales are utilized for achieved global feature maps to the adaptable average pool that is employed as priori details and additionally convolution, Rectified Linear Unit (ReLU) functions and batch normalization are accomplished for learning system parameters, capturing of local subarea’s feature information and decrease the size of the feature map. The learned subarea features with diverse scales are sampled and merged with actual feature maps. The cancer area segmented output obtained from PSPNet is implied by $P_{b}$ and architecture of PSPNet as demonstrated in Fig. 2.

#### Training of PSPNet utilizing JSO

JSO^31^ is a bio-enthused metaheuristic approach that is based on food-identifying characteristics of the jellyfish in the ocean. Due to the various applications of JSO, it is more suitable to train PSPNet for cancer area segmentation. While PSPNet typically includes its own training mechanism, in this research, JSO is employed as an alternative optimization strategy to enhance training effectiveness and accuracy.

##### Jellyfish search solution encoding

In $\rlap{--} z$ search space, the PSPNet learning parameter symbolized by $\Psi$ is tuned continually to achieve the best solution, such that $\rlap{--} z = \left[ {1 \times \Psi } \right]$.

##### Fitness function

A fitness measure is calculated by determining the difference between targeted and PSPNet output that can be illustrated by,

3 $$/\!\!\!\!F = \frac{1}{o}\sum\limits_{b = 1}^{o} {\left[ {{\rm T}_{b} - P_{b} } \right]\,^{2} }$$

where total image samples are signified by $o$ and targeted output is indicated by ${\rm T}_{b}$ whereas $P_{b}$ implies output from PSPNet.

The JSO follows the below-specified steps to achieve an excellent solution.

*Step 1: Initialize a solution*

A population of this approach is generally initialized randomly in $\rlap{--} z$ search space and it is represented as follows.

4 $$J = \left\{ {J_{1} ,J_{2} ,...,J_{s} ,...,J_{c} } \right\}$$

where $J_{c}$ specifies total variables, *s*th solution is symbolized by $J_{s}$ and $J$ implies population. JSO utilizes a chaotic map called a logistic map that gives many diverse initial populations than random choice and it results in a less probability of premature convergence, which can be given by,

5 $$J_{s + 1} = \beta \,J_{s} \,\left( {1 - J_{s} } \right),\,\,\,\,\,\,\,0 \le {/\!\!\!\!{J}}_{s} \le 1$$

Here, $J_{s + 1}$ denotes the position of *s*th jellyfish, ${/\!\!\!\!{J}}_{s}$ implies a logistic chaotic value of *s*th jellyfish position and $\beta$ signifies a parameter that is fixed to 4.

*Step 2: Estimating objective function*

An objective function is evaluated by computing variation amid PSPNet output and targeted output employing Eq. (3).

*Step 3: Following of ocean currents*

The ocean current comprises a larger amount of nutrients, thus jellyfish are fascinated towards it. The direction of the ocean current $\left( {\overrightarrow {R} } \right)$ is identified by taking an average of every vector from every jellyfish in the population to a jellyfish, which is present in a better position. It can be modeled by,

6 $$\overrightarrow {R} = J^{ * } - \eta \times r\,\left( {0,1} \right) \times \ell$$

Here $J^{ * }$ indicates jellyfish, which is presently in the finest position in a swarm, $\ell$ indicates the mean position of every jellyfish, $r$ signifies rand and $\eta$ specifies the distribution coefficient, which corresponds to ocean current length. The newer position of individual jellyfish can be illustrated as,

7 $$J_{s} \left( {\Im + 1} \right) = J_{s} \left( \Im \right) + r\left( {0,1} \right) \times \overrightarrow {R}$$

8 $$J_{s} \left( {\Im + 1} \right) = J_{s} \left( \Im \right) + r\left( {0,1} \right) \times J^{ * } - \eta \times r\left( {0,1} \right) \times \ell$$

Here, $J_{s} \left( \Im \right)$ signifies the present position of jellyfish whereas $J_{s} \left( {\Im + 1} \right)$ denotes the newer position of jellyfish.

*Step 4: Swarm of jellyfish*

In passive-type motion, the movement of jellyfish is across their individual positions. The related update position of individual jellyfish can be expressed by,

9 $$J_{s} \left( {\Im + 1} \right) = J_{s} \left( \Im \right) + \chi \times r\left( {0,1} \right) \times \left( {U - L} \right)$$

Here, $U$ illustrates the upper bound whereas $L$ denotes the lower bound and $\chi$ signifies the motion coefficient that corresponds to motion length across the individual location of jellyfish.

For simulating active type motion, the jellyfish $\left( q \right)$ other than one among interest is chosen randomly and a vector from a jellyfish of the interest $\left( s \right)$ to chosen jellyfish $\left( q \right)$ is utilized for determining movement direction. The motion direction and updated jellyfish location are formulated utilizing beneath equations.

10 $$\overrightarrow {S} = r\left( {0,1} \right) \times \overrightarrow {D}$$

11 $$\overrightarrow {D} = \left\{ \begin{gathered} J_{q} \left( \Im \right) - J_{s} \left( \Im \right),\,\,\,\,\,\,\,\,\,\,\,if\,{/\!\!\!\!{F}}\left( {J_{s} } \right) \ge {/\!\!\!\!{F}}\left( {J_{q} } \right) \hfill \\ J_{s} \left( \Im \right) - J_{q} \left( \Im \right),\,\,\,\,\,\,\,\,\,\,\,if\,{/\!\!\!\!{F}}\left( {J_{s} } \right) < {/\!\!\!\!{F}}\left( {J_{q} } \right) \hfill \\ \end{gathered} \right.$$

Therefore,

12 $$J_{s} \left( {\Im + 1} \right) = J_{s} \left( \Im \right) + \overrightarrow {S}$$

Here, ${/\!\!\!\!{F}}$ reveals the objective function of position $J$, $S$ denotes step and $D$ implies direction.

*Step 5: time control mechanism*

For regulating the motion of jellyfish amongst subsequent ocean currents as well as moving within jellyfish swarm, a time controlling mechanism contains the time-control function indicated as $\xi \left( \Im \right)$ as well as constant denoted by $x_{0}$. A time-control function is a randomly chosen value, which alters from 0 to 1 over time. It can be evaluated by,

13 $$\xi \left( \Im \right) = \left| {\,\left( {1 - \frac{\Im }{N}} \right) \times \left( {2 \times r\left( {0,1} \right) - 1} \right)\,} \right|$$

Here $\Im$ represents time, which is illustrated by iteration count whereas $N$ implies the maximal count of iterations.

*Step 6: Termination*

JSO is terminated by performing the above-elucidated steps until the best solution is acquired.

### Image augmentation

An image augmentation technique can considerably increase the count of samples. It is very precise as it is formerly produced from the ground truth naturally and this technique overcomes the overfitting issue. Here, data augmentation techniques like rotation, random erasing and flipping are considered. A segmented input $P_{b}$ is taken as input to perform image augmentation.

#### Rotation

Rotation^32^ is a traditional geometric image augmentation process that is executed by rotating an image in right or left directions by angles among 1 and 359. The rotation process is applied to an image by a certain degree of angles in a further manner. The equation to compute rotation is represented as,

14 $$A_{1} = \left[ \begin{gathered} \cos \,\varphi \,\, - \sin \,\varphi \hfill \\ \sin \,\varphi \,\,\,\,\,\,\,\cos \,\varphi \,\, \hfill \\ \end{gathered} \right]\,.\,\left[ \begin{gathered} d \hfill \\ h \hfill \\ \end{gathered} \right]$$

where $d$ and $h$ symbolize a pair of the coordinates whereas $\varphi$ signifies rotation angle.

#### Random erasing

The fundamental concept of random erasing^32^ is to erase a single square in the square area of an image randomly. A random erasing technique is proven as highly efficient and it is signified by $A_{2}$.

#### Flipping

The flipping^32^ technique reflects the image across the horizontal or vertical axis or both axes. Flipping assists the users in maximizing the count of images in the database without requiring artificial processing.

(i) Vertical flipping

Vertical flipping refers to the rotation of an image in an upside-down manner, therefore x-axis is on the bottom whereas the y-axis is presented on top. A vertical flipping can be modeled by,

15 $$Y_{1} = \left[ \begin{gathered} 1\,\,\,\,\,\,\,\,\,\,0 \hfill \\ 0\,\,\,\,\,\, - 1 \hfill \\ \end{gathered} \right]\,\,\left[ \begin{gathered} d \hfill \\ h \hfill \\ \end{gathered} \right]$$

(ii) Horizontal flipping

In horizontal flipping, an image is horizontally rotated to right as well as left sides that can be given as follows.

16 $$Y_{2} = \left[ \begin{gathered} - 1\,\,\,\,\,\,\,\,\,\,0 \hfill \\ \,\,\,\,0\,\,\,\,\,\,\,\,\,1 \hfill \\ \end{gathered} \right]\,\,\left[ \begin{gathered} d \hfill \\ h \hfill \\ \end{gathered} \right]$$

(iii) Horizontal and vertical flipping

This type of flipping specifies to horizontal and vertical rotation of an image, wherein both horizontal and vertical columns are conserved. The horizontal and vertical flipping can be represented as,

17 $$Y_{3} = \left[ \begin{gathered} - 1\,\,\,\,\,\,\,\,\,\,0 \hfill \\ \,\,\,\,0\,\,\,\,\,\, - 1 \hfill \\ \end{gathered} \right]\,\,\left[ \begin{gathered} d \hfill \\ h \hfill \\ \end{gathered} \right]$$

The flipping technique is denoted by $A_{3}$, in such a manner that,

18 $$A_{3} = \left\{ {Y_{1} ,Y_{2} ,Y_{3} } \right\}$$

The augmented output obtained is symbolized by $A_{b}$, such that it is expressed by,

19 $$A_{b} = \left\{ {A_{1} ,A_{2} ,A_{3} } \right\}$$

### Feature extraction

The BC issue intends to predict the properties of newer tumor (benign or malignant). In the feature extraction stage, abstract benign and malignant tumor patterns are extracted separately before the actual image is trained in the classifier to decrease the higher dimensionality of the feature spaces. For recognizing patterns, feature extraction is utilized. Here, an augmented image $A_{b}$ is taken to accomplish feature extraction. The features that are included for extraction are Gabor features, LBP, statistical features, shape features and LVP. Gabor features are effective for extracting texture and edge information at multiple orientations and scales, LBP captures local texture patterns that are useful for identifying subtle differences in tissue appearance, and shape features provide structural and boundary-related information critical for distinguishing tumor regions. Together, these features enhance the performance of the model to recognize complex patterns and contribute significantly to improving classification accuracy. 

#### Gabor features

Gabor features^33,34^ specified as Gabor bank, Gabor jet or a multi-resolution Gabor feature are created from the responses of Gabor filters by utilizing numerous filters on various frequencies as well as orientations. As a core of the Gabor filter enabled feature extraction is a two-dimensional Gabor filter operation, it can be modeled as,

20 $$T_{1} = \frac{{\rlap{--} f^{2} }}{\pi \omega \aleph }e^{{ - \left( {\frac{{\rlap{--} f^{2} }}{{\omega^{2} }}\rlap{--} x^{{\prime}{2}} + \frac{{\rlap{--} f^{2} }}{{\aleph^{2} }}\rlap{--} y^{{\prime}{2}} } \right)}} \,e^{{\rlap{--} j2\pi \rlap{--} f\rlap{--} x^{\prime}}}$$

21 $$\rlap{--} x^{\prime} = \rlap{--} x\,\cos \theta + \rlap{--} y\sin \theta$$

22 $$\rlap{--} y^{\prime} = \rlap{--} y\,\sin \theta + \rlap{--} y\cos \theta$$

where $\rlap{--} f$ indicates the central frequency of the filter, $\omega$ signifies sharpness or bandwidth along the Gaussian major axis, $\aleph$ denotes sharpness or bandwidth along the Gaussian minor axis and $\theta$ specifies rotation angle.

#### LBP

LBP^35^ is primitively designed for $3 \times 3$ neighbors, offering 8-bit codes on the basis of neighbor pixels over the middle one. For a pixel at $\left( {\rlap{--} m_{O} ,\rlap{--} n_{O} } \right)$, a resulting LBP can be illustrated by,

23 $$T_{2} = \sum\nolimits_{{\rlap{--} r = 0}}^{7} {{\rm I}\,\left( {\rlap{--} g_{{\rlap{--} k}} - \rlap{--} g_{O} } \right)\,2^{{\rlap{--} r}} }$$

Here, $\rlap{--} r$ denotes 8 neighborhoods of middle pixel whereas $\rlap{--} g_{O}$ and $\rlap{--} g_{{\rlap{--} k}}$ implies gray-level values of middle and adjacent pixels.

#### LVP

On the basis of vector depiction, the LVP^36^ descriptor is devised for providing several two-dimensional spatial architectures of micro-patterns with diverse pair-wise vector directions of reference pixels and its neighbors. LVP is represented as,

24 $$T_{3} = \left\{ {LVP_{{\Upsilon ,{\rm X},\hbar }} \left( {V_{E} } \right)\,\left| {\,\hbar = 0^{ \circ } ,\,45^{ \circ } ,\,90^{ \circ } ,\,135^{ \circ } } \right.} \right\}$$

Here, $V_{E}$ symbolizes to reference pixel.

From Gabor features, LBP and LVP, the texture feature image indicated as $Z_{b}$ is obtained, such that,

25 $$Z_{b} = \left\{ {T_{1} ,T_{2} ,T_{3} } \right\}$$

Then, shape features and the statistical features are applied to the texture feature image to obtain the feature vector.

#### Shape features

The shape features^37^ namely compactness, eccentricity, rectangularity and solidity are considered for extraction.

(i) Compactness

The compactness is a ratio of the area and perimeter of an image pixel that can be evaluated utilizing beneath equation.

26 $$H_{1} = \frac{{P_{r} }}{{A_{r} }}$$

where $P_{r}$ implies perimeter and $A_{r}$ signifies area.

(ii) Eccentricity

It is signified as a ratio of the major axis length to the minor axis length. It can be computed by the fundamental axis method or minimum bounding method. Eccentricity is formulated as shown below.

27 $$H_{2} = \frac{\rm M}{{\rm O}}$$

Here ${\rm M}$ symbolizes the major axis length and ${\rm O}$ represents the minor axis length.

(iii) Rectangularity

Rectangularity indicates a ratio amongst the area and the eccentricity values. It can be calculated as mentioned below.

28 $$H_{3} = \frac{{A_{r} }}{{H_{2} }}$$

where $H_{2}$ reveals eccentricity.

(iv) Solidity

Solidity refers to a degree of shape whether it is concave or convex and it is estimated utilizing the below equation.

29 $$H_{4} = \frac{{A_{r} }}{{C_{h} }}$$

Here $C_{h}$ denotes the region of the convex hull for an area in pixels.

The shape features are symbolized as $H_{b}$, such that it is illustrated by,

30 $$H_{b} = \left\{ {H_{1} ,H_{2} ,H_{3} ,H_{4} } \right\}$$

#### Statistical features

Standard deviation (SD), mean, kurtosis, variance and skewness are the statistical features^38^ concerned for extraction.

(i) Mean

Mean implies to arithmetical average of the group of values and mean can be mathematically formulated as,

31 $$Y_{1} = \sum\limits_{\vartheta = 0}^{{{\rm E} - 1}} {\vartheta * {\rm A}\left( \vartheta \right)}$$

where ${\rm A}\left( \vartheta \right)$ signifies the probability of $\vartheta$ occurrence, $\vartheta$ illustrates individual grey levels whereas ${\rm E}$ mentions the overall count of grey levels.

(ii) Variance

Variance indicates a deviation value of an image’s grey-level corresponding to the mean grey-level. It can be calculated by,

32 $$Y_{2} = \sum\limits_{\vartheta = 0}^{{{\rm E} - 1}} {\left( {\vartheta - Y_{1} } \right)^{2} * {\rm A}\left( \vartheta \right)}$$

Here $Y_{1}$ signifies mean.

(iii) SD

SD reveals data dispersion across a mean. The lower SD specifies that values are much closer to the mean whereas the higher SD shows a wider dispersion of values against the mean. The SD can be formulated by,

33 $$Y_{3} = \sqrt {\sum\limits_{\vartheta = 0}^{{{\rm E} - 1}} {\left( {\vartheta - Y_{1} } \right)^{2} * {\rm A}\left( \vartheta \right)} }$$

(iv) Skewness

Skewness is defined as a degree of asymmetry of the distribution of certain features across a mean. It may be negative or else positive values and SD is calculated as follows.

34 $$Y_{4} = \left( {Y_{1} } \right)^{ - 3} \left[ {\sum\limits_{\vartheta = 0}^{{{\rm E} - 1}} {\left( {\vartheta - Y_{1} } \right)^{3} * {\rm A}\left( \vartheta \right)} } \right]$$

(v) Kurtosis

Kurtosis calibrates a leveling of the distribution with respect to an actual distribution that is modeled by,

35 $$Y_{5} = \left( {Y_{1} } \right)^{ - 4} \left[ {\sum\limits_{\vartheta = 0}^{{{\rm E} - 1}} {\left( {\vartheta - Y_{1} } \right)^{4} * {\rm A}\left( \vartheta \right)} } \right]$$

The statistical features are mentioned as $Y_{b}$, in such a manner that,

36 $$Y_{b} = \left\{ {Y_{1} ,Y_{2} ,Y_{3} ,Y_{4} ,Y_{5} } \right\}$$

From a feature extraction stage, the feature vector $T_{b}$ is acquired, in such a way that,

37 $$T_{b} = \left\{ {H_{b} ,Y_{b} } \right\}$$

### BCD utilizing BCDCNN based on adaptive error similarity

BC is most common among females across the globe. It is considered as the most regularly diagnosed non-skin cancer and the primary cause of death due to cancer among women. BC is the second most unsafe cancer after lung cancer. Earlier detection can save the lives of people as it is easy to treat and prevent tumor from expansion. Here, BCD is carried out by BCDCNN on the basis of adaptive error similarity, wherein the extracted image $T_{b}$ is passed as the input to execute BCD. DCNN^39,40^ is considered one of the most expansively utilized ML approaches, particularly in vision-associated applications. The DCNN is capable to learn depictions from grid-based data and currently, it has been revealed considerable performance enhancement in several ML uses. The general CNN structure normally consists of alternative layers of convolutional (conv), pooling and one or multiple fully connected (FC) layers. In a few conditions, the FC layer is substituted by means of global average pooling layers. The BC-detected output from DCNN is revealed by $B_{b}$ .

#### BCDCNN based on adaptive error similarity

The loss function is newly designed based on adaptive error similarity^41^. In classification problems, let $M$ indicate sample space and limited group of labels is represented as ${/\!\!\!\!{L}}$, wherein ${/\!\!\!\!{L}} = \left\{ {\rlap{--} l_{1} ,\rlap{--} l_{2} ,...,\rlap{--} l_{\varpi } } \right\}$, $\varpi > 2$. Thus, the mapping association of the sample $z \in M^{{\rlap{--} N}}$ for labeling set ${/\!\!\!\!{L}}$ is several to one. The multi-class classification chore is to forecast sample classes from multi-classes.

(i) Gradient descent

The common neural network (NN) on the basis of softmax activation function comprises three layers namely input, output and hidden layers. A softmax function transforms numerical outcomes of NN to probability output. Furthermore, the predicted class is determined by maximal probability in output. Thereafter, cross-entropy (CE) is utilized for measuring the variation amongst true labels and predicted classes at individual training iteration. Afterwards, an error or variation is propagated back to a hidden layer for adjusting weights. Consider $g_{u,v}$ implies the association weight of *u*th neuron to *v*th neuron. Additionally, $G$ represents the weight matrix that is comprised of $g_{u,v}$ and $g_{u}$ reveals *u*th row vector of the matrix $G$. Hence, the output can be given by $a = G\,z$. However, output $a$ is not normalized. To resolve this issue, the softmax operation has gained considerable attention in a multi-class classification that is utilized be an output layer. An output layer of NN comprises $\varpi$ cells and individual cells correspond to the label. Furthermore, $t_{u}$ indicates *u*th coordinate value of vector $t$ whereas $\tilde{t}_{u}$ symbolizes *u*th coordinate value of a vector $\tilde{t}$. A softmax maps the output outcomes to intervals 0 and 1, wherein the sum of every output is 1. Hence, it converts classification trouble into probability form that gives intuitive options for the last decision. A softmax can be illustrated by,

38 $$t_{u} = \frac{{\exp \,\left( {a_{u} } \right)}}{{\sum\nolimits_{v = 1}^{\varpi } {\exp \,\left( {a_{v} } \right)} }}$$

Here, $u \in \left\{ {1,2,...,\varpi } \right\}$ and neural output is given by $a_{u} = \sum\nolimits_{v} {g_{uv} z_{uv} }$. Thus, it can be attained by,

39 $$\sum\limits_{u = 1}^{\varpi } {t_{u} } = 1$$

The major purpose of NN is tuning the weight matrix $G$. The weight values are adjusted by back-propagation of individual iteration errors. Furthermore, errors are produced by a loss function or else a cost function. Assume $f^{i} \left( G \right)$ represents loss function at the iteration $i$. Therefore, the optimization problem can be modeled by,

40 $$\min \sum\limits_{i = 1}^{I} {f^{i} \left( G \right)}$$

Here $I$ signifies the overall count of training iterations. To obtain an optimum solution, the optimization approaches search through the direction of gradients. In addition, the gradient of loss function at *i*th iteration while concerning *u*th output cell is mathematically expressed by,

41 $$\nabla f^{i} \left( {g_{u} } \right) = \frac{{\partial f^{i} \left( {g_{u} } \right)}}{{\partial g_{u} }}$$

Hence, an update model of a weight vector $g_{u}$ in the gradient descent-enabled approaches is formulated as shown beneath,

42 $$g_{u}^{i} = g_{u}^{i - 1} - \mu \,\nabla f^{i} \left( {g_{u}^{i - 1} } \right)$$

where $\mu$ indicates learning rate.

(ii) CE

CE loss function generated from the information theory that calibrates a loss amongst predicted and true distributions of existing techniques. In the following phase, the CE loss function as well as its gradient while utilizing a multi-classifier is formulated by,

43 $$f^{i} \left( G \right) = - \sum\limits_{u = 1}^{\varpi } {\tilde{t}_{u} } \,\log \,\left( {soft\max \,\left( {g_{u} z} \right)} \right)$$

44 $$f^{i} \left( G \right) = - \sum\limits_{u = 1}^{\varpi } {\tilde{t}_{u} } \,\log \,\left( {t_{u} } \right)$$

Here, $\varpi$ reveals the overall count of classes, $t_{u}$ implies *u*th prediction class and $\tilde{t}_{u}$ signifies *u*th true class of the training samples. From Eq. (41) and Eq. (44) and based on the chain rule,

45 $$\nabla f^{i} \left( {g_{u} } \right) = - \frac{{\partial f^{i} \left( {g_{u} } \right)}}{{\partial t_{v} }}\,\frac{{\partial t_{v} }}{{\partial a_{u} }}\,\frac{{\partial a_{u} }}{{\partial g_{u} }}$$

Here,

46 $$\frac{{\partial f^{i} \left( {g_{u} } \right)}}{{\partial t_{v} }} = \sum\nolimits_{v = 1}^{p} {\frac{{\partial \,\left( { - \tilde{t}_{v} \,\log \,t_{v} } \right)}}{{\partial t_{v} }}}$$

47 $$\frac{{\partial f^{i} \left( {g_{u} } \right)}}{{\partial t_{v} }} = - \sum\nolimits_{v = 1}^{p} {\tilde{t}_{v} } \frac{1}{{t_{v} }}$$

Also, $z \in M^{{\rlap{--} N}}$ is normalized prior to the training procedure, thus it can be given by,

48 $$\frac{{\partial a_{u} }}{{\partial g_{u} }} = \sum\limits_{v = 1}^{p} {z_{uv} = 1}$$

Here, $p$ implies the overall count of association units of the cell $u$. Consider two conditions of association units such as $u = v$ and $u \ne v$. If $u = v$, then,

49 $$\frac{{\partial t_{u} }}{{\partial a_{u} }} = \frac{{\partial \,\left( {\frac{{\exp \,\left( {a_{u} } \right)}}{{\sum\nolimits_{p = 1}^{\varpi } {\exp \,\left( {a_{p} } \right)} }}} \right)}}{{\partial a_{u} }}$$

50 $$\frac{{\partial t_{u} }}{{\partial a_{u} }} = t_{u} \,\left( {1 - t_{u} } \right)$$

Moreover, if $u \ne v$, then,

51 $$\frac{{\partial t_{u} }}{{\partial a_{u} }} = \frac{{\partial \,\left( {\frac{{\exp \,\left( {a_{uv} } \right)}}{{\sum\nolimits_{\alpha = 1}^{\varpi } {\exp \,\left( {a_{u\alpha } } \right)} }}} \right)}}{{\partial a_{u} }}$$

52 $$\frac{{\partial t_{u} }}{{\partial a_{u} }} = - t_{u} t_{v}$$

Applying Eq. (45) to Eq. (52), the obtained equation can be modeled by,

53 $$\nabla f^{i} \left( {g_{u} } \right) = - \left( {\sum\limits_{v = 1}^{p} {\tilde{t}_{v} \frac{1}{{t_{v} }}} } \right)\,\frac{{\partial t_{v} }}{{\partial a_{u} }}$$

54 $$\nabla f^{i} \left( {g_{u} } \right) = t_{u} \sum\limits_{v} {\tilde{t}_{v} } - t_{u}$$

Also, in accordance to Eq. (39) and Eq. (54), the equation can be represented by,

55 $$\nabla f^{i} \left( {g_{u} } \right) = t_{u} - \tilde{t}_{u}$$

(iii) Conditional autoregressive value-at-risk (CAViaR) and CE-based stochastic gradient

Let $t_{\max }$ specifies maximal in $\left\{ {t_{1} ,t_{2} ,...,t_{\varpi } } \right\}$, wherein $\varpi$ indicates the count of classes and $\varepsilon$ th class implies actual class. Furthermore, $\varepsilon$ th coordinate of $\tilde{t}$ is 1. Consider $t^{\prime}: = \left( {t_{\max } - t_{\varepsilon } } \right)\,\tilde{t}$ having $\tilde{t}$ that signifies vector of actual classes. Owing to the values of the unreal classes are 0, the equation can be given by,

56 $$\sum\limits_{u = 1}^{\varpi } {\left( {t_{\max } - t_{u} } \right)\,\tilde{t}_{u} = t_{\max } - t_{\varepsilon } }$$

Thus, the maximized CE loss function can be illustrated by,

57 $$f^{\prime}\left( G \right) = - \sum\limits_{u = 1}^{\varpi } {t_{u}{\prime} \,\log \,\left( {t_{u} } \right)}$$

58 $$f^{\prime}\left( G \right) = - \sum\limits_{u = 1}^{\varpi } {\left( {t_{\max } - t_{\varepsilon } } \right)\,\tilde{t}_{u} \log \,\left( {t_{u} } \right)}$$

Here, $t_{u}{\prime}$ implies $u^{th}$ coordinate value of the vector $t^{\prime}$. In accordance to Eq. (41) and Eq. (58), the equation can be expressed by,

59 $$\frac{{\partial f^{i} \left( {g_{u} } \right)}}{{\partial t_{v} }} = \frac{{\partial \,\left( { - \sum\nolimits_{v} {\left( {t_{\max } - t_{v} } \right)\,\tilde{t}_{u} \,\log \,t_{v} } } \right)}}{{\partial t_{v} }}$$

60 $$\frac{{\partial f^{i} \left( {g_{u} } \right)}}{{\partial t_{v} }} = - \sum\limits_{v} {\left( {t_{\max } - t_{v} } \right)\,\frac{{\tilde{t}_{u} }}{{t_{v} }}}$$

From Eq. (45), Eq. (52), Eq. (54), Eq. (56) and Eq. (60), the equation attained is represented as,

61 $$\nabla f^{\prime}\left( {g_{u} } \right) = \left( { - \sum\limits_{v} {\left( {t_{\max } - t_{v} } \right)\,\frac{{\tilde{t}_{u} }}{{t_{v} }}} } \right)\,\frac{{\partial t_{v} }}{{\partial a_{u} }}$$

62 $$\nabla f^{\prime}\left( {g_{u}^{i - 1} } \right) = t_{u} \,\left( {t_{\max } - t_{\varepsilon } } \right) - \left( {t_{\max } - t_{u} } \right)\,\tilde{t}_{u}$$

Substitute Eq. (62) in Eq. (42),

63 $$g_{u}^{i} = g_{u}^{i - 1} - \mu \,\left( {t_{u} \,\left( {t_{\max } - t_{\varepsilon } } \right) - \left( {t_{\max } - t_{u} } \right)\,\tilde{t}_{u} } \right)$$

Let us consider,

64 $$t_{u} = t_{u}^{i - 1}$$

Substitute the above consideration in Eq. (63), then the equation becomes,

65 $$g_{u}^{i} = g_{u}^{i - 1} - \mu \,\left( {t_{u}^{i - 1} \,\left( {t_{\max } - t_{\varepsilon } } \right) - \left( {t_{\max } - t_{u}^{i - 1} } \right)\,\tilde{t}_{u} } \right)$$

66 $$g_{u}^{i - 1} = g_{u}^{i} + \mu \,\left( {t_{u}^{i - 1} \,\left( {t_{\max } - t_{\varepsilon } } \right) - \left( {t_{\max } - t_{u}^{i - 1} } \right)\,\tilde{t}_{u} } \right)$$

The standard equation from CAViaR^42^ is formulated by,

67 $$g_{u}^{i} = \phi_{0} + \sum\limits_{l = 1}^{j} {\phi_{l} * g_{u}^{i - \varpi } } + \sum\limits_{m = 1}^{\gamma } {\phi_{m} \,F\,\left( {g_{u}^{i - m} } \right)}$$

Consider, $j = \gamma = 2$, then the above equation becomes,

68 $$g_{u}^{i} = \phi_{0} + \phi_{1} \,g_{u}^{i - 1} + \phi_{2} \,g_{u}^{i - 2} + \phi_{1} \,F\,\left( {g_{u}^{i - 1} } \right) + \phi_{2} \,F\,\left( {g_{u}^{i - 2} } \right)$$

Substitute Eq. (68) in Eq. (66),

69 $$g_{u}^{i - 1} = \phi_{0} + \phi_{1} \,g_{u}^{i - 1} + \phi_{2} \,g_{u}^{i - 2} + \phi_{1} \,F\,\left( {g_{u}^{i - 1} } \right) + \phi_{2} \,F\,\left( {g_{u}^{i - 2} } \right) + \mu \,\left( {t_{u}^{i - 1} \,\left( {t_{\max } - t_{\varepsilon } } \right) - \left( {t_{\max } - t_{u}^{i - 1} } \right)\,\tilde{t}_{u} } \right)$$

where

70 $$\phi_{1} = \frac{{e_{i} }}{{e_{i - 1} }}$$

71 $$\phi_{2} = \frac{{e_{i} }}{{e_{i - 2} }}$$

Here, $\phi_{0}$ represents a constant and $e$ indicates an error.

## Results and discussion

BCDCNN for BCD achieved excellent outcomes for the assessments performed and thus acquired outcomes are elucidated in beneath segment.

### Experimentation setup

To perform BCD, BCDCNN designed in this research is experimentally performed in PYTHON tool.

### Description of dataset

The datasets utilized to accomplish BCD are Breast Cancer Patients MRI’s and Dynamic contrast-enhanced magnetic resonance images of breast cancer patients with tumor locations (Duke-Breast-Cancer-MRI).

#### Breast cancer patients MRI’s

In the Breast Cancer Patients MRI’s dataset^26^, the images are classified into two categorizations such as sick or malignant and healthy or benign. For training purposes, both classifications comprise 700 MRI images of sick and healthier patients. For the purpose of validation, both categorizations comprise 40 MRI images of sick and healthier patients.

#### Duke-breast-cancer-MRI

Duke-Breast-Cancer-MRI dataset^27^ is a single-organized, retrospective accumulation of about 922 biopsy affirmed invasive BC patients for a decade. It comprises 922 participants, 922 studies, 5161 series and 773,888 images of about 368.4 GB.

#### CBIS-DDSM: breast cancer image dataset

The CBIS-DDSM dataset^43^ comprises 10.2 k JPG images and 6 CSV files, totaling 6.3 GB. It contains 103 columns, among them 65 string type, 29 integer-type, 4 ID fields, and 5 of other types. This dataset supports research in breast cancer detection and classification using mammography images.

### Experimentation outcomes

**Fig. 3:**
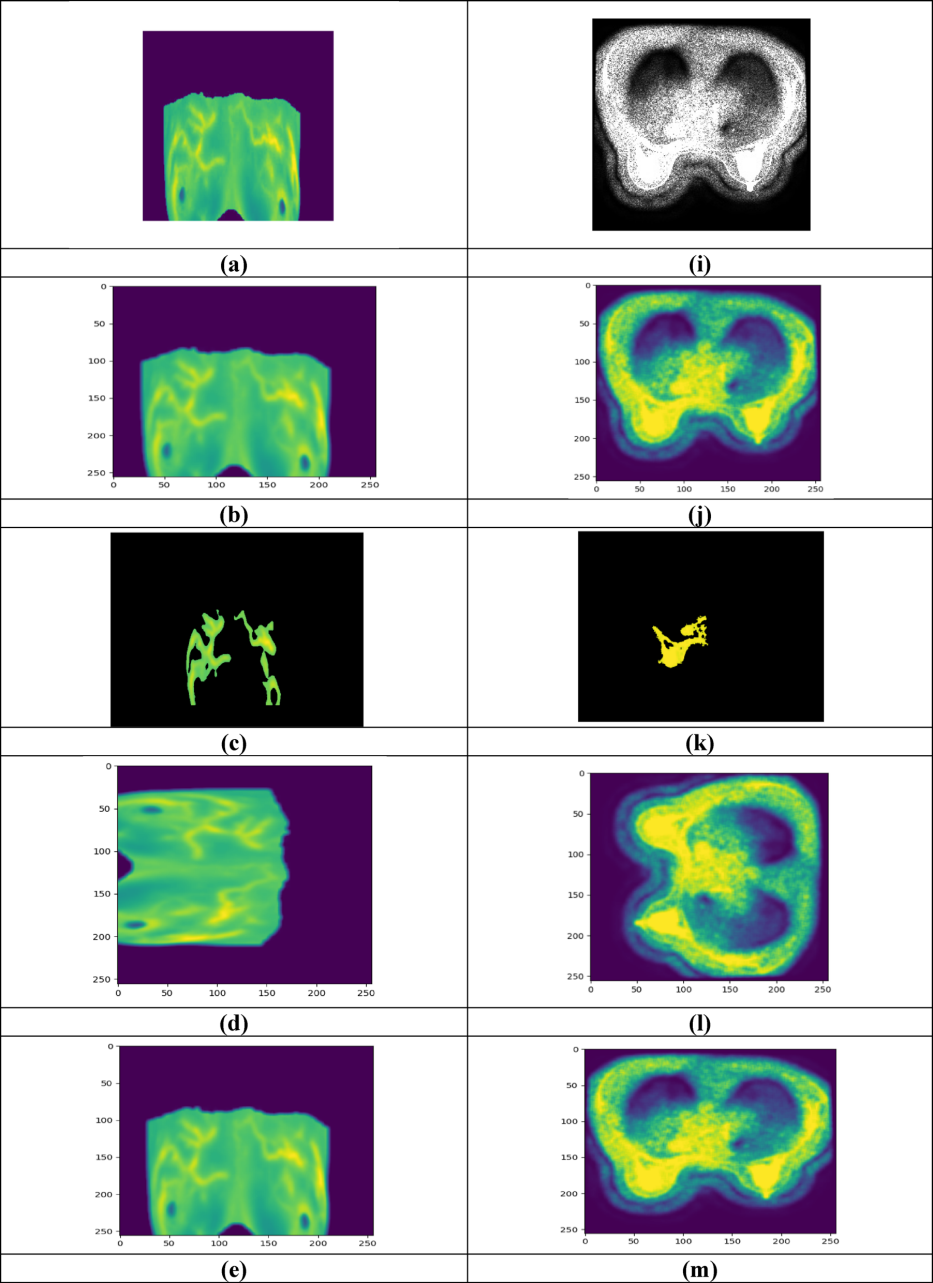

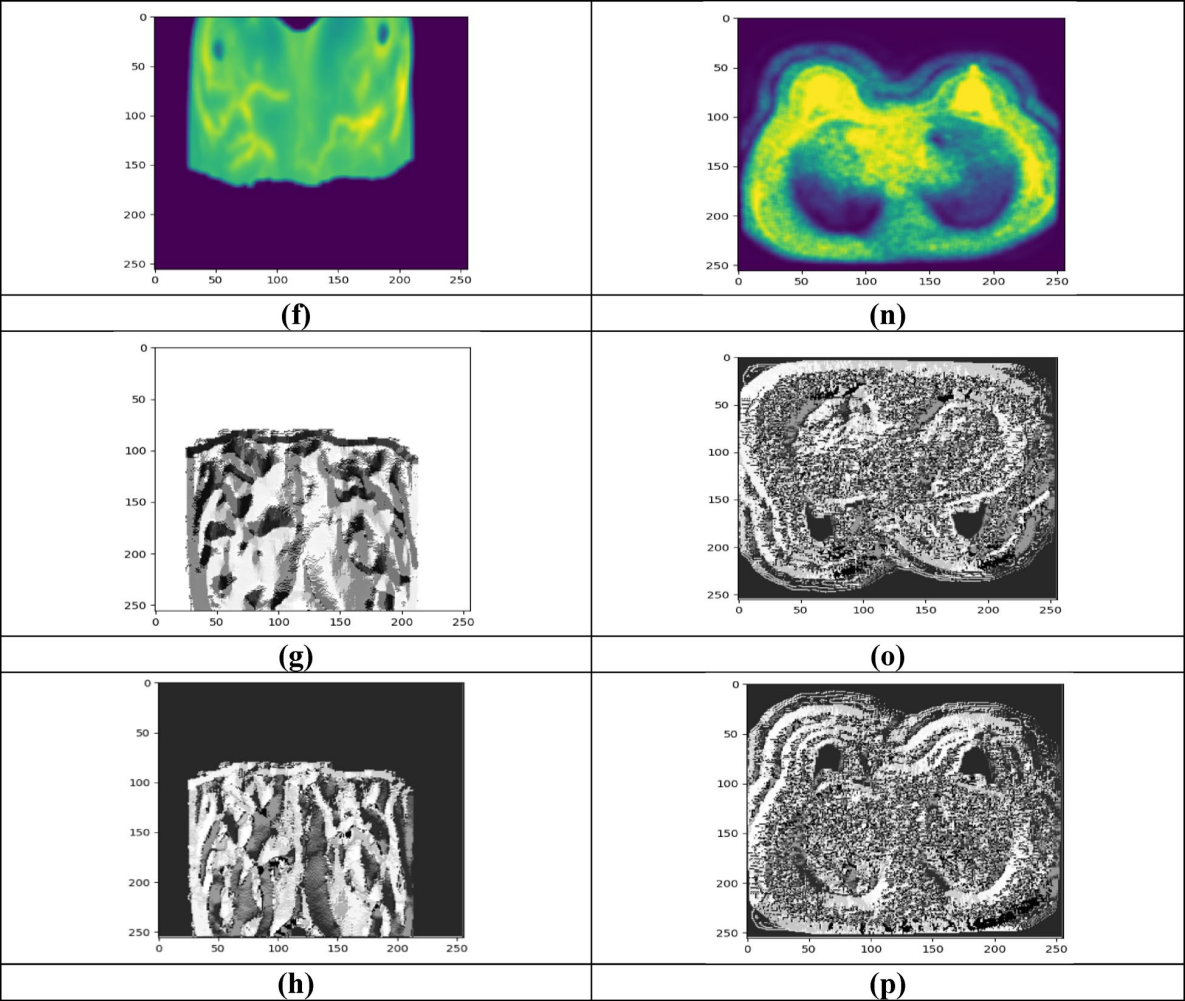
Experimentation outcomes, (**a**) Input image-1, (**b**) Filtered image-1, (**c**) Segmented image-1, (**d**) Rotated image-1, (**e**) Random erased image-1, (**f**) Flipped image-1, (**g**) LBP image-1, (**h**) LVP image-1, (**i**) input image-2, (**j**) filtered image-2, (**k**) segmented image-2, (**l**) rotated image-2, (**m**) random erased image-2, (**n**) flipped image-2, (**o**) LBP image-1, (**p**) LVP image-2.

Figure 3 displays the experimentation outcomes of BCDCNN for two datasets. Figure 3a–h shows input image-1, filtered image-1, segmented image-1, rotated image-1, random erased image-1, flipped image-1, LBP image-1 and LVP image-1 for Breast Cancer Patients MRI’s dataset whereas input image-2, filtered image-2, segmented image-2, rotated image-2,—random erased image 2, flipped image-2, LBP image-1 and LVP image-2 for Duke-Breast-Cancer-MRI dataset is demonstrated in Fig. 3i–p.

### Performance measures

Sensitivity, specificity and accuracy are performance measures considered for the estimation of BCDCNN for BCD.

#### Accuracy

Accuracy specifies a ratio of precisely classified samples to overall samples that are calculated as follows,

72 $$\Re_{1} = \frac{{Tr_{pt} + Tr_{nt} }}{{Tr_{pt} + Tr_{nt} + Fa_{pt} + Fa_{nt} }}$$

where $Tr_{pt}$ and $Tr_{nt}$ indicates true positive as well as true negative whereas $Fa_{pt}$ and $Fa_{nt}$ mentions false positive and false negative.

#### Sensitivity

Sensitivity refers to a rate of perceived positive cases with overall positive cases, which is given by,

73 $$\Re_{2} = \frac{{Tr_{pt} }}{{Tr_{pt} + Fa_{nt} }}$$

#### Specificity

Specificity is referred as an association of viewed negative instances with every negative instance. Also, it is the rate of detected occurrence including complete instances by an occurrence of BC. Sensitivity can be formulated by,

74 $$\Re_{3} = \frac{{Tr_{nt} }}{{Tr_{nt} + Fa_{pt} }}$$

### Comparative techniques

AlexNet^15^, CNN^6^, DL^11^, Gaussian SVM^22^, DLA-EABA^4^, and Ultrafast DCE-MRI^23^ are the conventional approaches concerned to assess with BCDCNN to show its effectiveness.

### Comparative evaluation

A comparative evaluation of BCDCNN is accomplished regarding Breast Cancer Patients MRI’s and Duke-Breast-Cancer-MR datasets with concern to evaluation metrics.

#### Assessment based on breast cancer patients MRI’s DATASET

BCDCNN is evaluated by changing training data and K value with regard to three performance metrics.

(i) Evaluation based on data

**Fig. 4:**
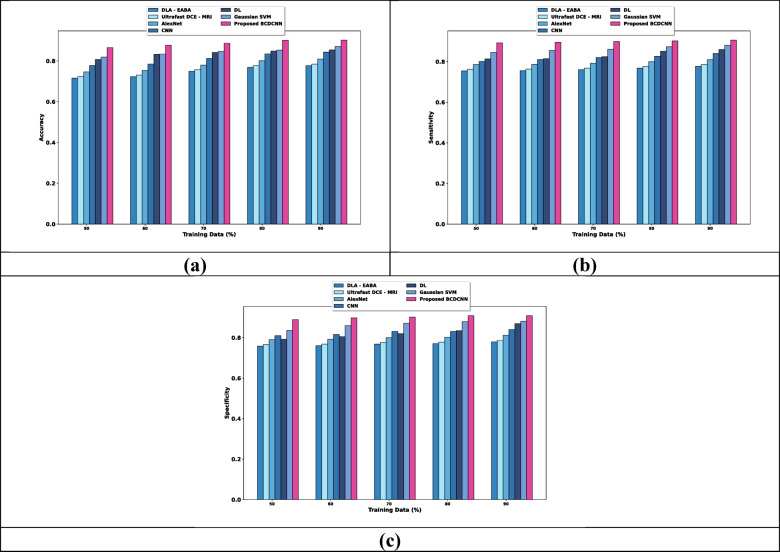
Comparative estimation regarding training data for Breast Cancer Patients MRI’s dataset, (**a**) Accuracy, (**b**) Sensitivity, (**c**) Specificity.

Figure 4 elucidates the evaluation of BCDCNN concerning three metrics by changing training data. For training, data = 90%, the accuracy, sensitivity and specificity values obtained by BCDCNN and traditional techniques are interpreted. Analysis of BCDCNN relating to accuracy is revealed in Fig. 4a. Accuracy acquired by BCDCNN is 0.902 whereas DLA-EABA, Ultrafast DCE-MRI, AlexNet, CNN, DL and Gaussian SVM obtained 0.777, 0.785, 0.809, 0.843, 0.854 and 0.871 that shows performance enhancement about 13.858%, 12.971%, 10.320%, 6.583%, 5.354% and 3.434%. Figure 4b mentions the evaluation of BCDCNN regarding sensitivity. BCDCNN attained a sensitivity value of 0.906 while the sensitivity obtained by DLA-EABA is 0.778, Ultrafast DCE-MRI is 0.786, AlexNet is 0.810, CNN is 0.840, DL is 0.860 and Gaussian SVM is 0.881. This explains the improvement of performance about 14.128%, 13.245%, 10.606%, 7.329%, 5.145% and 2.803%. Estimation of BCDCNN considering specificity is described in Fig. 4c. DLA-EABA, Ultrafast DCE-MRI, AlexNet, CNN, DL and Gaussian SVM achieved a specificity of 0.780, 0.788, 0.812, 0.841, 0.869 and 0.881 whereas BCDCNN obtained 0.909. The enhancement of performance by BCDCNN is 14.191%, 13.311%, 10.755%, 7.533%, 4.463% and 3.131%.

(ii) Evaluation based on K value

**Fig. 5:**
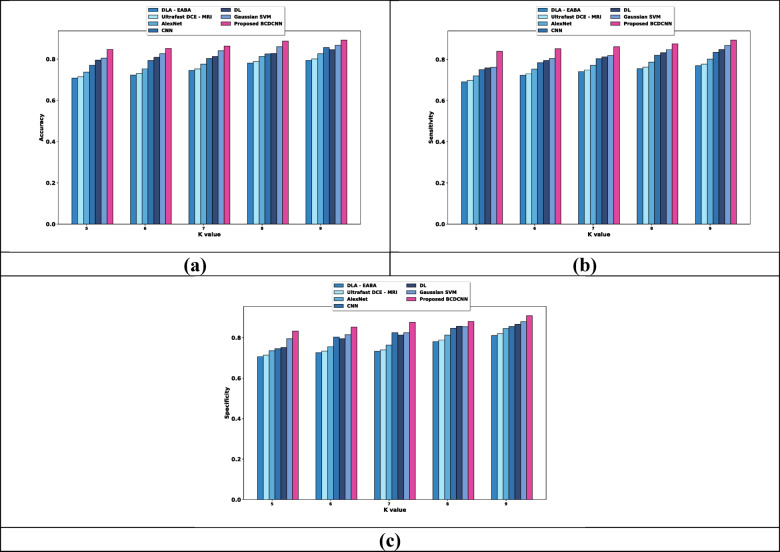
Comparative assessment regarding K-value for Breast Cancer Patients MRI’s dataset, (**a**) Accuracy, (**b**) Sensitivity, (**c**) Specificity.

Analysis of BCDCNN with regard to measures by changing K values is delineated in Fig. 5. When the K value is 9, the values acquired by BCDCNN and traditional approaches are explicated. Figure 5a indicates the estimation of BCDCNN with respective to accuracy. The value of accuracy acquired by BCDCNN is 0.892 while accuracy attained by DLA-EABA is 0.793, Ultrafast DCE-MRI is 0.801, AlexNet is 0.826, CNN is 0.856, DL is 0.846 and Gaussian SVM is 0.867. Assessment of BCDCNN in respect to sensitivity is illustrated in Fig. 5b. Sensitivity acquired by BCDCNN is 0.922 whereas DLA-EABA, Ultrafast DCE-MRI, AlexNet, CNN, DL and Gaussian SVM obtained 0.778, 0.786, 0.810, 0.840, 0.860, 0.881, and 0.906. Figure 5c specifies the evaluation of BCDCNN concerning specificity. The specificity values obtained by DLA-EABA, Ultrafast DCE-MRI, AlexNet, CNN, DL and Gaussian SVM are 0.811, 0.820, 0.845, 0.855, 0.866, and 0.879 while BCDCNN achieved 0.909 that describes 10.781%, 9.791%, 7.041%, 5.941%, 4.730%, and 3.300% of performance improvement by BCDCNN.

#### Assessment based on duke-breast-cancer-MR dataset

The analysis of BCDCNN is done in relation to considered evaluation metrics by altering training data and K value.

(i) Evaluation based upon training data

**Fig. 6:**
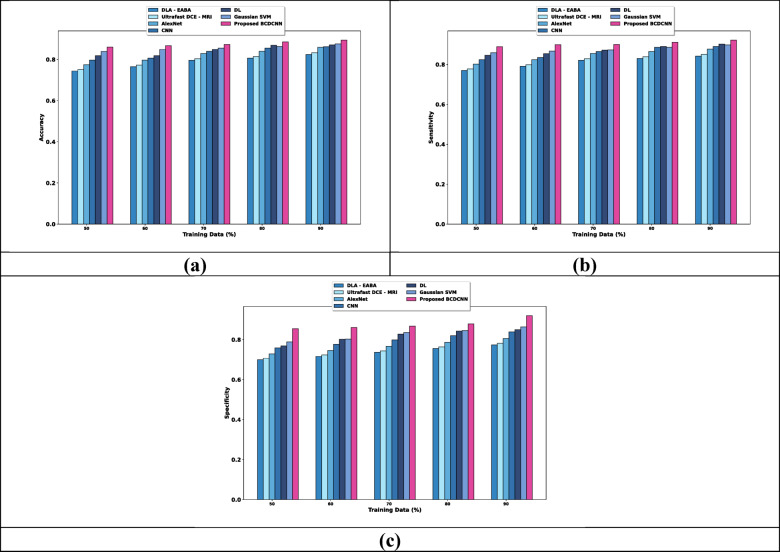
Comparative assessment regarding training data for Duke-Breast-Cancer-MR dataset, (**a**) Accuracy, (**b**) Sensitivity, (**c**) Specificity.

Figure 6 interprets the estimation of BCDCNN regarding metrics by altering training data. When considered training data is 90%, the values of evaluation metrics achieved by BCDCNN and conventional methods are expounded. Evaluation of BCDCNN in respect to accuracy is shown in Fig. 6a. DLA-EABA, Ultrafast DCE-MRI, AlexNet, CNN, DL and Gaussian SVM obtained values of accuracy about 0.825, 0.833, 0.859, 0.862, 0.871, and 0.876 while BCDCNN acquired 0.894. Assessment of BCDCNN in regard to sensitivity is explicated in Fig. 6b. The sensitivity value attained by BCDCNN is 0.922 whereas DLA-EABA, Ultrafast DCE-MRI, AlexNet, CNN, DL and Gaussian SVM acquired 0.842, 0.851, 0.877, 0.890, 0.902, and 0.898. Figure 6c displays an analysis of BCDCNN with concerning specificity. BCDCNN obtained a specificity of 0.920 while values acquired by DLA-EABA is 0.774, Ultrafast DCE-MRI is 0.782, AlexNet is 0.806, CNN is 0.839, DL is 0.850 and Gaussian SVM is 0.864.

(ii) Evaluation in terms of K value

**Fig. 7:**
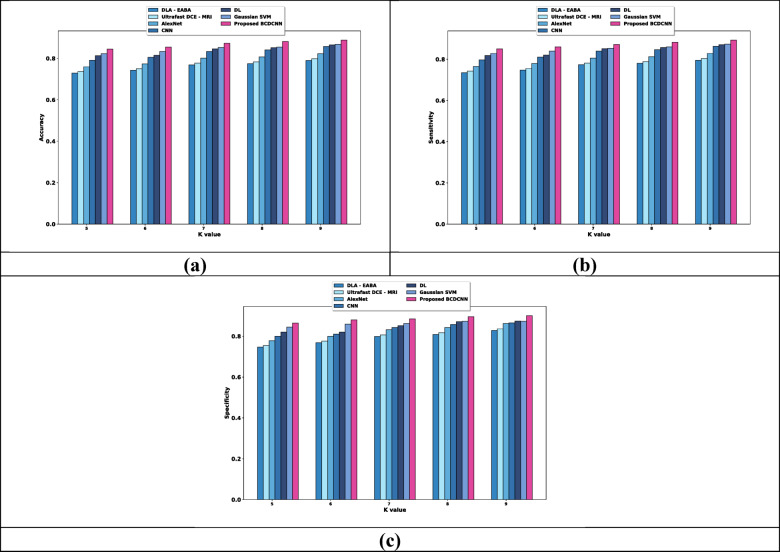
Comparative estimation regarding K value for Duke-Breast-Cancer-MR dataset, (**a**) Accuracy, (**b**) Sensitivity, (**c**) Specificity.

Estimation of BCDCNN concerning measures by changing K values is illustrated in Fig. 7. The values attained by BCDCNN and conventional techniques for K value = 9 are described. Analysis of BCDCNN in terms of accuracy is displayed in Fig. 7a. BCDCNN achieved an accuracy of 0.888 while DLA-EABA, Ultrafast DCE-MRI, AlexNet, CNN, DL and Gaussian SVM achieved 0.790, 0.798, 0.823, 0.857, 0.865, and 0.868. Figure 7b reveals an assessment of BCDCNN based upon sensitivity. The sensitivity values acquired by DLA-EABA, Ultrafast DCE-MRI, AlexNet, CNN, DL and Gaussian SVM are 0.795, 0.804, 0.829, 0.863, 0.871, and 0.874 whereas BCDCNN obtained 0.893. Figure 7c delineates the estimation of BCDCNN by means of specificity. The value of specificity attained by BCDCNN is 0.901 while specificity achieved by DLA-EABA is 0.828, Ultrafast DCE-MRI is 0.836, AlexNet is 0.862, CNN is 0.865, DL is 0.874 and Gaussian SVM is 0.873.

#### Assessment based on CBIS-DDSM: breast cancer image dataset

The analysis of BCDCNN is done in relation to considered evaluation metrics by altering training data using CBIS-DDSM: Breast Cancer Image Dataset.

(i) Evaluation based upon training data

**Fig. 8:**
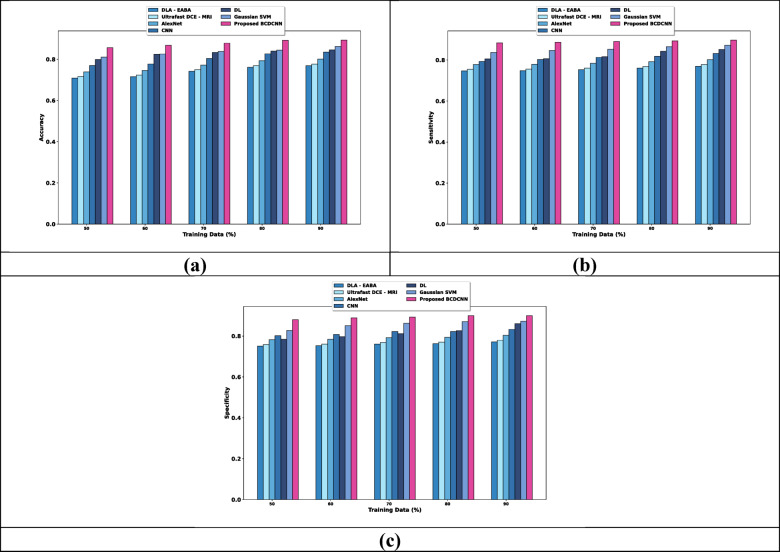
Comparative assessment regarding training data for CBIS-DDSM: Breast Cancer Image Dataset, (**a**) Accuracy, (**b**) Sensitivity, (**c**) Specificity.

The estimation of BCDCNN regarding metrics by altering training data is provided in Fig. 8. When considered training data is 90%, the values of evaluation metrics are expounded. Evaluation of accuracy is shown in Fig. 8a. DLA-EABA, Ultrafast DCE-MRI, AlexNet, CNN, DL and Gaussian SVM obtained values of accuracy about 0.769, 0.777, 0.801, 0.835, 0.845, and 0.862 while BCDCNN acquired 0.893. Assessment of BCDCNN in regard to sensitivity is explicated in Fig. 8b. The sensitivity value attained by BCDCNN is 0.897 whereas DLA-EABA, Ultrafast DCE-MRI, AlexNet, CNN, DL and Gaussian SVM acquired 0.770, 0.778, 0.802, 0.832, 0.851, and 0.872. Figure 8c displays an analysis of BCDCNN with concerning specificity. BCDCNN obtained a specificity of 0.900 while values acquired by DLA-EABA is 0.772, Ultrafast DCE-MRI is 0.780, AlexNet is 0.804, CNN is 0.833, DL is 0.860 and Gaussian SVM is 0.872.

### Scalability analysis

**Fig. 9:**
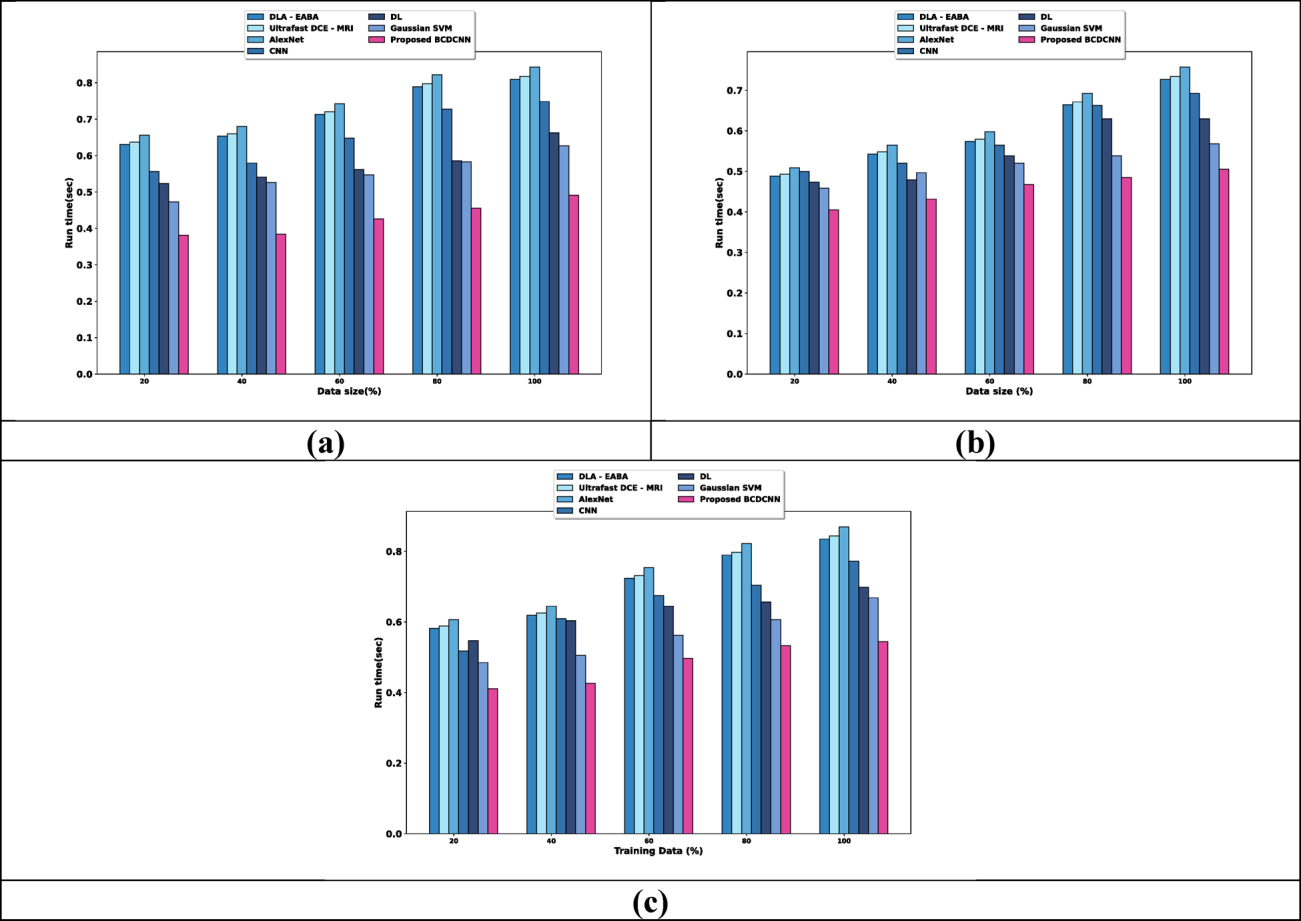
Scalability analysis using, (**a**) Breast Cancer Patients MRI’s dataset, (**b**) Duke-Breast-Cancer-MR dataset, (**c**) CBIS-DDSM: Breast Cancer Image Dataset.

The estimation of BCDCNN regarding metrics by altering training data is provided in Fig. 9. The scalability analysis using Breast Cancer Patients MRI’s dataset is shown in Fig. 9a. DLA-EABA, Ultrafast DCE-MRI, AlexNet, CNN, DL and Gaussian SVM required runtime of 0.809 s, 0.818 s, 0.843 s, 0.748 s, 0.662 s, and 0.627 s while BCDCNN required minimum runtime of 0.491 s, for datasize 100. The scalability assessment of BCDCNN using the Duke-Breast-Cancer-MR dataset is explicated in Fig. 9b. Runtime required by BCDCNN is 0.485 s whereas DLA-EABA, Ultrafast DCE-MRI, AlexNet, CNN, DL and Gaussian SVM required runtime of 0.664 s, 0.671 s, 0.692 s, 0.662 s, 0.630 s, and 0.538 s, for datasize = 80. Figure 9c displays analysis of BCDCNN using CBIS-DDSM: Breast Cancer Image Dataset. BCDCNN needed the runtime of 0.544 s, while runtime values acquired by DLA-EABA is 0.835 s, Ultrafast DCE-MRI is 0.843 s, AlexNet is 0.870 s, CNN is 0.772 s, DL is 0.698 s and Gaussian SVM is 0.668 s, for datasize = 100.

### Computational time analysis

**Fig. 10:**
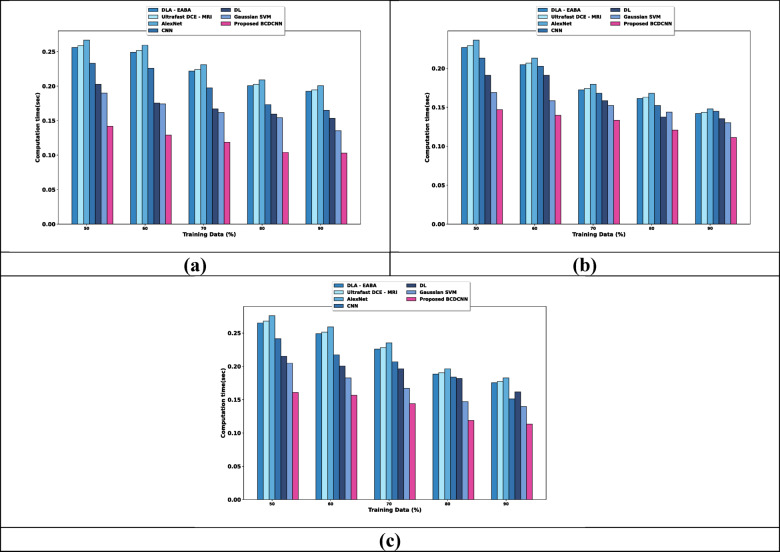
Computational time analysis using, (**a**) Breast Cancer Patients MRI’s dataset, (**b**) Duke-Breast-Cancer-MR dataset, (**c**) CBIS-DDSM: Breast Cancer Image Dataset.

The computational time analysis for three datasets is depicted in Fig. 10. When considered training data is 90%, the computational time required for the models is discussed in this section. The computational time evaluation using the Breast Cancer Patients MRI’s dataset is discussed in Fig. 10a. DLA-EABA, Ultrafast DCE-MRI, AlexNet, CNN, DL and Gaussian SVM required the values of computational time of 0.193 s, 0.195 s, 0.201 s, 0.165 s, 0.153 s, and 0.135 s, while BCDCNN required 0.103 s. Assessment of computational time using the Duke-Breast-Cancer-MR dataset is explicated in Fig. 10b. The computational time value required by BCDCNN is 0.111 s whereas DLA-EABA, Ultrafast DCE-MRI, AlexNet, CNN, DL and Gaussian SVM required 0.142 s, 0.144 s, 0.148 s, 0.145 s, 0.135 s, and 0.130 s. Figure 10c displays an analysis of computational time using CBIS-DDSM: Breast Cancer Image Dataset. BCDCNN obtained a computational time of 0.113 s while the computational time needed by DLA-EABA is 0.175 s, Ultrafast DCE-MRI is 0.177 s, AlexNet is 0.183 s, CNN is 0.151 s, DL is 0.162 s and Gaussian SVM is 0.140 s.

### Analysis based on memory

**Fig. 11:**
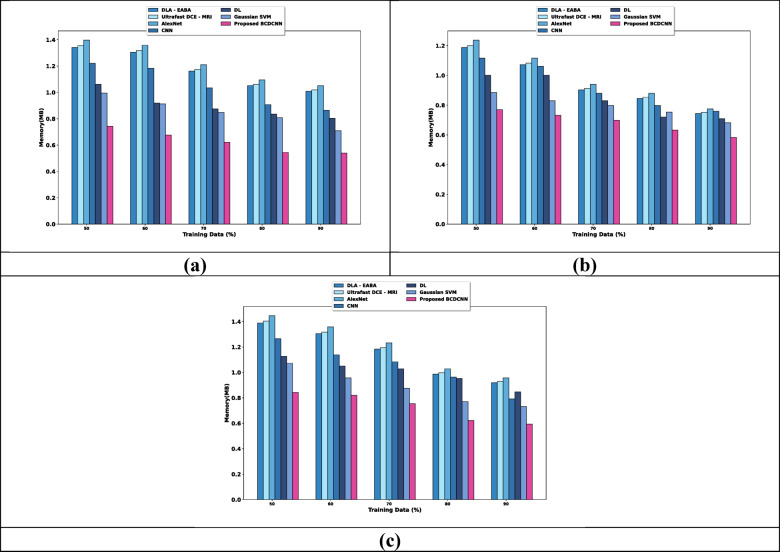
Analysis based on memory using, (**a**) Breast Cancer Patients MRI’s dataset, (**b**) Duke-Breast-Cancer-MR dataset, (**c**) CBIS-DDSM: Breast Cancer Image Dataset.

The analysis based on memory for three datasets is discussed in Fig. 11. When considered training data is 90%, the memory required for the models is discussed in this section. The memory evaluation using the Breast Cancer Patients MRI’s dataset is discussed in Fig. 11a. DLA-EABA, Ultrafast DCE-MRI, AlexNet, CNN, DL and Gaussian SVM required the values of memory of 1.008 MB, 1.019 MB, 1.051 MB, 0.864 MB, 0.803 MB, and 0.710 MB, while BCDCNN required 0.539 MB. Assessment of memory using the Duke-Breast-Cancer-MR dataset is explicated in Fig. 11b. Memory value required by BCDCNN is 0.583 MB whereas DLA-EABA, Ultrafast DCE-MRI, AlexNet, CNN, DL and Gaussian SVM required 0.744 MB, 0.752 MB, 0.776 MB, 0.759 MB, 0.710 MB, and 0.682 MB. Figure 11c displays an analysis of memory using CBIS-DDSM: Breast Cancer Image Dataset. BCDCNN obtained memory of 0.594 MB while memory needed by DLA-EABA is 0.919 MB, Ultrafast DCE-MRI is 0.928 MB, AlexNet is 0.957 MB, CNN is 0.792 MB, DL is 0.847 MB and Gaussian SVM is 0.732 MB.

#### Training and validation curve

**Fig. 12:**
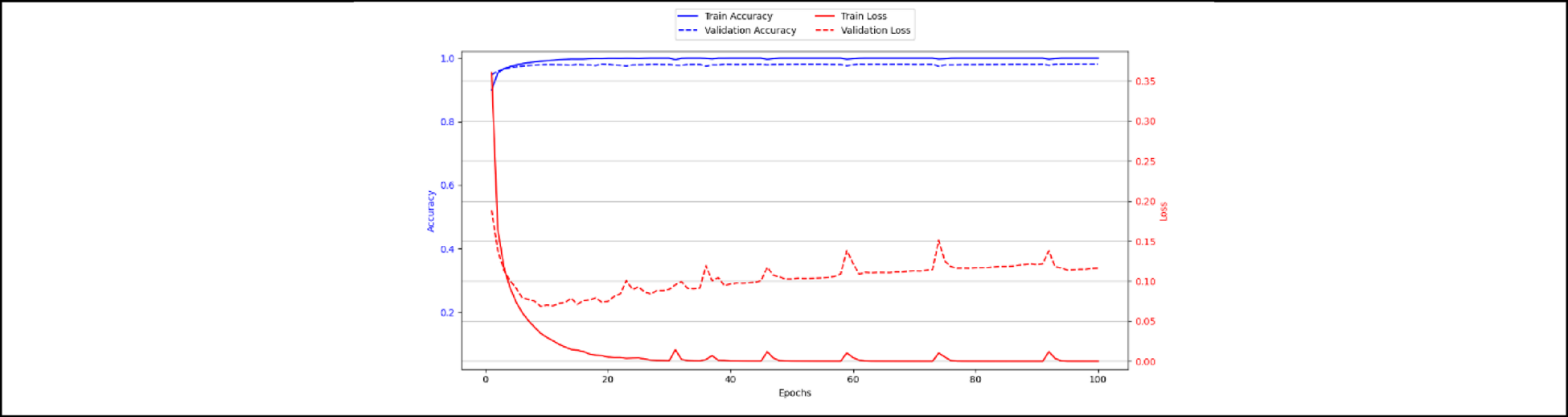
Training and validation curve.

Figure 12 shows the training and validation accuracy and loss of the model over 100 epochs. The blue lines represent accuracy, while the red lines represent loss. From the graph, the training accuracy quickly reaches nearly 100% and stays constant, which means the model is learning the training data very well. Similarly, the training loss drops close to zero, confirming that the model is performing perfectly on the training set. The validation curve is shown in red dashed line. It decreases in the beginning, and it starts to increase slightly and shows fluctuations after a certain point. The validation accuracy is shown in the blue dashed line, which is slightly lower than the training accuracy.

#### Comparative discussion

BCDCNN designed for BCD obtained the best outcomes while comparing with AlexNet, CNN, DL and Gaussian SVM. Table 2 specifies the discussion table with values obtained for assessments by BCDCNN and traditional approaches. It is recognized that BCDCNN acquired maximum values of accuracy, sensitivity and specificity about 90.2%, 90.6% and 90.9% when training data = 90% for Breast Cancer Patients MRI’s dataset. Thus, the results clearly reveal that BCDCNN is an excellent approach to performing BCD to decrease the mortality rate and increase the patient’s survival rate.

**Table 2:** Comparative discussion of BCDCNN.

Analysis based upon	Metrics/Methods	DLA-EABA	Ultrafast DCE-MRI	AlexNet	CNN	DL	Gaussian SVM	Proposed BCDCNN
Breast Cancer Patients MRI’s dataset								
Training data = 90%	Accuracy (%)	77.7	78.5	80.9	84.3	85.4	87.1	**90.2**
	Sensitivity (%)	77.8	78.6	81.0	84.0	86.0	88.1	**90.6**
	Specificity (%)	78.0	78.8	81.2	84.1	86.9	88.1	**90.9**
K value = 9	Accuracy (%)	79.3	80.1	82.6	85.6	84.6	86.7	89.2
	Sensitivity (%)	77.0	77.8	80.2	83.5	84.9	86.8	89.4
	Specificity (%)	81.1	82.0	84.5	85.5	86.6	87.9	90.9
Duke-Breast-Cancer-MR dataset								
Training data = 90%	Accuracy (%)	82.5	83.3	85.9	86.2	87.1	87.6	89.4
	Sensitivity (%)	84.2	85.1	87.7	89.0	90.2	89.8	92.2
	Specificity (%)	77.4	78.2	80.6	83.9	85.0	86.4	92.0
K value = 9	Accuracy (%)	79.0	79.8	82.3	85.7	86.5	86.8	88.8
	Sensitivity (%)	79.5	80.4	82.9	86.3	87.1	87.4	89.3
	Specificity (%)	82.8	83.6	86.2	86.5	87.4	87.3	90.1
CBIS-DDSM: Breast Cancer Image Dataset								
Training data = 90%	Accuracy (%)	76.9	77.7	80.1	83.5	84.5	86.2	89.3
	Sensitivity (%)	77.0	77.8	80.2	83.2	85.1	87.2	89.7
	Specificity (%)	77.2	78.0	80.4	83.3	86.0	87.2	90.0

#### Statistical analysis

To evaluate the statistical significance of the performance differences among the compared methods, an Analysis of Variance (ANOVA) test is conducted, as presented in Table 3. The ANOVA results show that the sum of squares for the treatments is 0.04213 with 3 degrees of freedom, while the residual sum of squares is 0.032548 with 120 degrees of freedom. The F-statistic value is calculated as 20.025418, and the associated p-value is 2.335E^-11^, which is significantly lower than the standard threshold of 0.05. This indicates that there is a statistically significant difference between at least one pair of the compared methods, confirming that the performance improvements achieved by the proposed BCDCNN model are statistically meaningful.

**Table 3:** Statistical test using ANOVA.

	Sum of squares	Degrees of freedom	F	P-value
C (treatments)	0.04213	3	20.025418	2.335E^−11^
Residual	0.032548	120		

## Conclusion

A second kind of cancer by which many women die throughout the world is referred to as BC. If prediction is carried out in the starting stage of cancer, women have a better possibility of heal. Accurate detection of BC is an important and deadly issue in clinical society. In past decades, many researchers are attracted towards BC detection problems. In this research, a new approach termed BCDCNN is designed for BCD. The MRI image taken as input from the database is initially fed to perform pre-processing. AKF is utilized to filter an input image, which is then subjected to cancer area segmentation. PSPNet is employed to segment cancer areas in filtered images and PSPNet is tuned using JSO. Then, the image is augmented by considering augmentation techniques like flipping, random erasing and rotation. Afterwards, features namely LBP, statistical features, Gabor features, LVP and shape features are extracted. Finally, BCD is executed by BCDCNN in which the loss function is devised on the basis of adaptive error similarity. Furthermore, designed BCDCNN attained high accuracy, sensitivity and specificity values of about 90.2%, 90.6% and 90.9% for considered training data = 90% for Breast Cancer Patients MRI’s dataset. However, the proposed model tends to overfit when trained on small datasets and may show reduced performance on data from different sources. In addition, it requires high computational power, which limits its use on low-resource devices. Future improvements will focus on enhancing the model’s ability to handle larger and more diverse datasets. Integration of clinical information with image data is also planned to improve overall diagnostic accuracy.

## Electronic supplementary material

Below is the link to the electronic supplementary material.

Supplementary Material 1

## Data Availability

The datasets generated during and/or analysed during the current study are available in the Breast Cancer Patients MRI’s at https://www.kaggle.com/datasets/uzairkhan45/breast-cancer-patients-mris, Duke-Breast-Cancer-MRI at https://wiki.cancerimagingarchive.net/pages/viewpage.action?pageId=70226903, and CBIS-DDSM: Breast Cancer Image Dataset at https://www.kaggle.com/datasets/awsaf49/cbis-ddsm-breast-cancer-image-dataset.

## Code availability

The source code of this research is available at https://github.com/MartinaaJ/BCDCNN.git.

## References

[1] Ozmen, V. Breast cancer in the world and Turkey. *J. Breast Health* **4**(2), 6–12 (2008).

[2] Tabar, L. et al. Mammography service screening and mortality in breast cancer patients: 20-year follow-up before and after introduction of screening. *The Lancet* **361**(9367), 1405–1410 (2003). 10.1016/S0140-6736(03)13143-1 12727392

[3] Sheth, D. & Giger, M. L. Artificial intelligence in the interpretation of breast cancer on MRI. *J. Magn. Reson. Imaging* **51**(5), 1310–1324 (2020). 31343790 10.1002/jmri.26878

[4] Zheng, J. et al. Deep learning assisted efficient AdaBoost algorithm for breast cancer detection and early diagnosis. *IEEE Access* **8**, 96946–96954 (2020).

[5] Gøtzsche, P. C. & Jørgensen, K. J. Screening for breast cancer with mammography. *Cochrane Database Syst. Rev.* **6**, 1877 (2013). 10.1002/14651858.CD001877.pub5 PMC6464778 23737396

[6] Yurttakal, A. H., Erbay, H., İkizceli, T. & Karaçavuş, S. Detection of breast cancer via deep convolution neural networks using MRI images. *Multimedia Tools Appl.* **79**, 15555–21557 (2020).

[7] Saslow, D. et al. American Cancer Society guidelines for breast screening with MRI as an adjunct to mammography. *CA* **57**(2), 75–89 (2007). 17392385 10.3322/canjclin.57.2.75

[8] Mann, R. M., Cho, N. & Moy, L. Breast MRI: State of the art. *Radiology* **292**(3), 520–536 (2019). 31361209 10.1148/radiol.2019182947

[9] Hassan, I., Umar, M. & Dokoro, A. H. An innovative prototype for diagnosing and treatment of breast cancer: A case study of specialist hospital Gombe. *Multimedia Res.* **5**(2), 1–6 (2022).

[10] Kuhl, C. The current status of breast MR imaging part I. Choice of technique, image interpretation, diagnostic accuracy, and transfer to clinical practice. *Radiology* **244**(2), 356–378 (2007). 17641361 10.1148/radiol.2442051620

[11] Eskreis-Winkler, S. et al. Using deep learning to improve nonsystematic viewing of breast cancer on MRI. *J. Breast Imaging* **3**(2), 201–207 (2021). 38424820 10.1093/jbi/wbaa102

[12] Hamidinekoo, A., Denton, E., Rampun, A., Honnor, K. & Zwiggelaar, R. Deep learning in mammography and breast histology: An overview and future trends. *Med. Image Anal.* **47**, 45–67 (2018). 29679847 10.1016/j.media.2018.03.006

[13] Brattain, L. J., Telfer, B. A., Dhyani, M., Grajo, J. R. & Samir, A. E. Machine learning for medical ultrasound: Status, methods, and future opportunities. *Abdom. Radiol.* **43**, 786–799 (2018). 10.1007/s00261-018-1517-0 PMC5886811 29492605

[14] Khoshdel, V., Ashraf, A. & LoVetri, J. Enhancement of multimodal microwave-ultrasound breast imaging using a deep-learning technique. *Sensors* **19**(18), 4050 (2019). 31546925 10.3390/s19184050 PMC6767656

[15] Nasir, M. U. et al. Breast cancer prediction empowered with fine-tuning. *Comput. Intell. Neurosci.* **2022**, 5918686 (2022). 35720929 10.1155/2022/5918686 PMC9203172

[16] Shah, D., Ullah Khan, M. A. & Abrar, M. Reliable breast cancer diagnosis with deep learning: DCGAN-driven mammogram synthesis and validity assessment. *Appl. Comput. Intell. Soft Comput.* **2024**(1), 1122109 (2024).

[17] Shah, D., Khan, M. A., Abrar, M. & Tahir, M. optimizing breast cancer detection with an ensemble deep learning approach. *Int. J. Intell. Syst.* **2024**(1), 5564649 (2024).

[18] Fernandis, J. R. ALOA: Ant lion optimization algorithm-based deep learning for breast cancer classification. *Multimedia Res.* **4**(1), 32–43 (2021).

[19] Choi, J. H. et al. Early prediction of neoadjuvant chemotherapy response for advanced breast cancer using PET/MRI image deep learning. *Sci. Rep.* **10**(1), 21149 (2020). 33273490 10.1038/s41598-020-77875-5 PMC7712787

[20] Yadav, A. R. & Kumar, V. N. PSO-optimized fractional order CNNs for enhanced breast cancer detection. *Results Eng.* **26**, 104559 (2025).

[21] LeCun, Y., Bengio, Y. & Hinton, G. Deep learning. *Nature* **521**(7553), 436–444 (2015). 26017442 10.1038/nature14539

[22] Daimiel Naranjo, I. et al. Radiomics and machine learning with multiparametric breast MRI for improved diagnostic accuracy in breast cancer diagnosis. *Diagnostics* **11**(6), 919 (2021). 34063774 10.3390/diagnostics11060919 PMC8223779

[23] Onishi, N. et al. Ultrafast dynamic contrast-enhanced breast MRI may generate prognostic imaging markers of breast cancer. *Breast Cancer Res.* **22**, 1–13 (2020). 10.1186/s13058-020-01292-9 PMC7254650 32466799

[24] Hu, Q., Whitney, H. M. & Giger, M. L. A deep learning methodology for improved breast cancer diagnosis using multiparametric MRI. *Sci. Rep.* **10**(1), 10536 (2020). 32601367 10.1038/s41598-020-67441-4 PMC7324398

[25] Bilal, A. et al. A quantum-optimized approach for breast cancer detection using SqueezeNet-SVM. *Sci. Rep.* **15**(1), 3254 (2025). 39863687 10.1038/s41598-025-86671-y PMC11763032

[26] Breast Cancer Patients MRI’s dataset is taken from “https://www.kaggle.com/datasets/uzairkhan45/breast-cancer-patients-mris”. Accessed on August 2023.

[27] Dynamic contrast-enhanced magnetic resonance images of breast cancer patients with tumor locations (Duke-Breast-Cancer-MRI) dataset is taken from, “https://wiki.cancerimagingarchive.net/pages/viewpage.action?pageId=70226903”. Accessed on August 2023.

[28] Rutan, S. C. Adaptive kalman filtering. *Anal. Chem.* **63**(22), 1103A-1109A (1991).

[29] Zhao, H., Shi, J., Qi, X., Wang, X. & Jia, J. Pyramid scene parsing network. In *Proceedings of the IEEE Conference on Computer Vision and Pattern Recognition*, pp. 2881–2890 (2017).

[30] Yang, C. & Guo, H. A method of image semantic segmentation based on pspnet. *Math. Probl. Eng.* **2022**, 8958154 (2022).

[31] Chou, J. S. & Molla, A. Recent advances in use of bio-inspired jellyfish search algorithm for solving optimization problems. *Sci. Rep.* **12**(1), 19157 (2022). 36357444 10.1038/s41598-022-23121-z PMC9649712

[32] Khalifa, N. E., Loey, M. & Mirjalili, S. A comprehensive survey of recent trends in deep learning for digital images augmentation. *Artif. Intell. Rev.* **55**, 2351–2377 (2022). 34511694 10.1007/s10462-021-10066-4 PMC8418460

[33] Shejul, A. A., Kinage, K. S. & Reddy, B. E. Gabor feature extraction driven facial age estimation using multilayer perceptron neural network. *Indian J. Comput. Sci. Eng. (IJCSE)* **11**(3) (2020).

[34] Kamarainen, J. K. Gabor features in image analysis. In *Proceedings of 2012 3rd International Conference on Image Processing Theory, Tools and Applications (IPTA)*, IEEE, pp.13–14 (2012).

[35] NarainPonraj, D., Christy, E., Aneesha, G., Susmitha, G. & Sharu, M. Analysis of LBP and LOOP based textural feature extraction for the classification of CT Lung images. In *Proceedings of 2018 4th International Conference on Devices, Circuits and Systems (ICDCS)*, pp. 309–312, IEEE, March (2018).

[36] Fan, K. C. & Hung, T. Y. A novel local pattern descriptor—Local vector pattern in high-order derivative space for face recognition. *IEEE Trans. Image Process.* **23**(7), 2877–2891 (2014). 24808409 10.1109/TIP.2014.2321495

[37] Kasim, A. A., Wardoyo, R. & Harjoko, A. Batik classification with artificial neural network based on texture-shape feature of main ornament. *Int. J. Intell. Syst. Appl.* **9**(6), 55 (2017).

[38] Lessa, V. & Marengoni, M. Applying artificial neural network for the classification of breast cancer using infrared thermographic images. In *Proceedings of Computer Vision and Graphics: International Conference, ICCVG 2016, Warsaw, Poland, September 19–21, 2016, Proceedings* 8, pp. 429–438. Springer International Publishing (2016).

[39] Khan, A., Sohail, A., Zahoora, U. & Qureshi, A. S. A survey of the recent architectures of deep convolutional neural networks. *Artif. Intell. Rev.* **53**, 5455–5516 (2020).

[40] Tu, F. et al. Deep convolutional neural network architecture with reconfigurable computation patterns. *IEEE Trans. Very Large Scale Integr. VLSI Syst.* **25**(8), 2220–2233 (2017).

[41] Zhou, Y. et al. MPCE: A maximum probability based cross entropy loss function for neural network classification. *IEEE Access* **7**, 146331–146341 (2019).

[42] Engle, R. F. & Manganelli, S. CAViaR: Conditional autoregressive value at risk by regression quantiles. *J. Bus. Econ. Stat.* **22**(4), 367–381 (2004).

[43] CBIS-DDSM: Breast Cancer Image Dataset taken from, “https://www.kaggle.com/datasets/awsaf49/cbis-ddsm-breast-cancer-image-dataset. Accessed on May 2025.

