# Supplementary material for: BCDCNN: breast cancer deep convolutional neural network for breast cancer detection using MRI images
Source: Sci Rep. 2025 Aug 8;15:29014. doi: 10.1038/s41598-025-09974-0 (PMC12334629; doi:10.1038/s41598-025-09974-0)
Supplement: Supplementary file 1 — Supplementary Material 1 [file 41598_2025_9974_MOESM1_ESM.docx]

Manuscript Title: **BCDCNN: Breast Cancer Deep Convolutional Neural Network for breast cancer detection using MRI images**

Manuscript ID: **47065ebf-2a41-44c3-86bc-fe614fe91e67 v1.0**

We utilized the Breast Cancer Patient MRI dataset, which is available on Kaggle (free online dataset). <https://www.kaggle.com/datasets/uzairkhan45/breast-cancer-patients-mris> . The link to access the dataset is provided in reference [25]. Dynamic contrast-enhanced magnetic resonance images of breast cancer patients with tumor locations (Duke-Breast-Cancer-MRI) dataset is taken from, “https://wiki.cancerimagingarchive.net/pages/viewpage.action? The link to access the dataset is provided in reference [26].
